# The TSP-1 domain of the matricellular protein CCN5 is essential for its nuclear localization and anti-fibrotic function

**DOI:** 10.1371/journal.pone.0267629

**Published:** 2022-04-27

**Authors:** Min Ho Song, Yongjoon Jo, Young-Kook Kim, Hyun Kook, Dongtak Jeong, Woo Jin Park

**Affiliations:** 1 College of Life Sciences, Gwangju Institute of Science and Technology, Gwangju, South Korea; 2 Department of Biochemistry, Chonnam National University Medical School, Hwasun, Jeollanam-do, Republic of Korea; 3 Department of Pharmacology, Chonnam National University Medical School, Hwasun, Jeollanam-do, Republic of Korea; 4 Department of Molecular & Life Science, College of Science and Convergence Technology, Hanyang University-ERICA, Ansan, Gyeonggi-do, Republic of Korea; University of Wisconsin-Madison, UNITED STATES

## Abstract

The matricellular protein CCN5 exerts anti-fibrotic activity in hearts partly by inducing reverse trans-differentiation of myofibroblasts (MyoFBs) to fibroblasts (FBs). CCN5 consists of three structural domains: an insulin-like growth factor binding protein (IGFBP), a von Willebrand factor type C (VWC), and a thrombospondin type 1 (TSP-1). In this study, we set out to elucidate the roles of these domains in the context of the reverse trans-differentiation of MyoFBs to FBs. First, human cardiac FBs were trans-differentiated to MyoFBs by treatment with TGF-β; this was then reversed by treatment with recombinant human CCN5 protein or various recombinant proteins comprising individual or paired CCN5 domains. Subcellular localization of these recombinant proteins was analyzed by immunocytochemistry, cellular fractionation, and western blotting. Anti-fibrotic activity was also evaluated by examining expression of MyoFB-specific markers, α-SMA and fibronectin. Our data show that CCN5 is taken up by FBs and MyoFBs mainly via clathrin-mediated endocytosis, which is essential for the function of CCN5 during the reverse trans-differentiation of MyoFBs. Furthermore, we showed that the TSP-1 domain is essential and sufficient for endocytosis and nuclear localization of CCN5. However, the TSP-1 domain alone is not sufficient for the anti-fibrotic function of CCN5; either the IGFBP or VWC domain is needed in addition to the TSP-1 domain.

## Introduction

The matricellular protein CCN5, also known as WNT1-inducible signaling pathway protein 2 (WISP-2), is a member of the cell communication network (CCN) family of proteins that regulate diverse cell behaviors such as proliferation, differentiation, and migration [1–9]. Previously, we demonstrated that CCN5 inhibits cardiac fibrosis (CF) partly by counteracting the pro-fibrotic activity of CCN2, also known as connective tissue growth factor (CTGF) [10]. Furthermore, we showed that CCN5 can reverse pre-established CF, in which two mechanisms are thought to be involved: 1) CCN5 induces apoptosis in myofibroblasts (MyoFBs) but not in fibroblasts (FBs) and cardiomyocytes (CMs); and 2) CCN5 induces reverse trans-differentiation of MyoFBs to FBs [11]. However, the molecular mechanism underlying the anti-fibrotic activity of CCN5 is yet to be fully understood.

CCN5 consists of three distinctive structural domains: an insulin-like growth factor binding protein (IGFBP), a von Willebrand factor type C (VWC), and a thrombospondin type 1 (TSP-1). In other CCN proteins (CCN1-4 and CCN6), these domains interact with diverse cytokines and integrins [12–15]. However, the roles of these domains in CCN5 are largely unknown. Previous studies suggested that CCN5 exerts its function in the nucleus [16, 17]. For example, CCN5 functions as a transcriptional co-repressor that down-regulates expression of the TGF-β receptor II in human tumor breast tissues [16]. Therefore, it was hypothesized that CCN5 is secreted from source cells via its amino-terminal signal sequence and is subsequently taken up by neighboring target cells via endocytosis (paracrine mode), after which it enters the nucleus and functions as a transcriptional co-activator or co-repressor. This hypothesis is yet to be tested vigorously.

In this study, we firstly tested whether exogenously-added CCN5 is translocated to nucleus in cardiac FBs and MyoFBs. We then examined which of the three domains plays an essential for the nuclear translocation of CCN5. We finally tested whether the nuclear translocation of CCN5 is critical for its anti-fibrotic activity. Human cardiac FBs was trans-differentiated to MyoFBs by treatment with TGF-β; this process was then reversed by treatment with purified human CCN5 protein. This *in vitro* setting was utilized as a read-out for the function of CCN5 or its domains throughout this study. In addition to the full-length CCN5 protein, we purified and tested recombinant proteins comprising individual domains (IGFBP, VWC, or TSP-1) or pairs of domains (VWC-TSP-1, IGFBP-TSP-1, or IGFBP-VWC) of CCN5. Our data indicate that CCN5 is indeed translocated to nucleus in cardiac FBs and MyoFBs and that the TSP-1 domain is essential for endocytosis and nuclear translocation of CCN5. In addition, the TSP-1-containing pairs of domains, VWC-TSP-1 and IGFBP-TSP-1, but not IGFBP-VWC, reversed trans-differentiation of MyoFBs to FBs. Collectively, these results suggest that the TSP-1 domain-mediated nuclear translocation is critical for the anti-fibrotic activity of CCN5.

## Material and methods

### Cell culture

Normal human ventricular cardiac FBs (Lonza, #CC-2904) were cultured in FGM-3 medium (Lonza, #CC-4526) at 37°C in a 5% CO_2_ incubator. HEK 293-F cells (Gibco, #R79007) were suspension-cultured in Freestyle 293 medium (Gibco, #12338018) at 37°C in a 8% CO_2_ incubator with shaking.

### Purification of recombinant proteins

cDNAs encoding the full-length human CCN5 protein, and individual domains (IGFBP, VWC, and TSP-1), and pairs of domains (VWC-TSP-1, IGFBP-TSP-1, and IGFBP-VWC) of human CCN5, were subcloned into a pcDNA3.1-myc-his plasmid and then transfected into HEK 293-F cells. One day before transfection, 1.5 × 10^7^ cells were seeded into 25 mL of Freestyle 293 medium. On the day of transfection, 20 μg of plasmid DNA was diluted in 3 mL of Opti-MEM medium (Gibco, #51985034). Next, 25 μl FectoPRO transfection reagent (Polyplus, #116–001) was added to the diluted plasmid DNA and the mixture was incubated for 10 mins at room temperature before being added gently to cultured HEK 293-F cells. Three days after transfection, the culture medium was collected and centrifuged at 2,000 × g for 3 mins. The proteins contained in culture medium were purified using Capturem His-Tagged Purification Maxiprep Columns (Takara, 635715). The purified proteins were stored in a buffer (20 mM NaHPO_4_, 150 mM NaCl and 250 mM imidazole, and 10% (v/v) glycerol) at -70°C.

### Trans-differentiation of FBs and protein treatment

Culture medium was changed to FBM (Lonza, #CC-3131) that did not contain any growth factors or serum. Next, FBs were treated with 10 ng/mL TGF-β (PeproTech, #100-35B) for 48 hours at 37°C in a 5% CO_2_ incubator. Finally, 20 nM of purified CCN5 protein was added to cells for 48 hours.

### Inhibition of endocytosis

MyoFBs were pre-incubated for 30 mins at 37°C with 60 nM phenylarsine oxide (PAO) (Sigma-Aldrich, P3075) and 1 mM methyl-β-cyclodextrin (MβCD) (Sigma-Aldrich, C4555). After pre-incubation with the endocytosis inhibitors, 20 nM of purified CCN5 protein was added to cells for 48 hours. Cells were washed by PBS before harvesting.

### Immunocytochemistry

Approximately, 15,000 FBs were seeded onto a 16 mm cover slip. Trans-differentiation of FBs to MyoFBs was induced by exposure to TGF-β2 for 48 hours at 37°C under 5% CO_2_. Cells were fixed with 4% paraformaldehyde, permeabilized with 0.5% Triton X-100, and blocked with 5% BSA solution. Next, cells were treated with antibodies against Myc (Abcam, ab32072) and α-SMA (Sigma-Aldrich, A5228), prior to incubation with secondary antibodies conjugated to Alexa Fluor 488 (Invitrogen, A11008) or Alexa Fluor 594 (Invitrogen, A11032). Hoechst dye was used to stain nuclei. Immunofluorescence signals were analyzed using ZEISS Axio Imager D2.

### Western blotting

RIPA buffer (50 mM Tris, 150 mM NaCl, 0.1% SDS, 1% Triton X-100, pH 8.0) was used to solubilize cell lysates containing Protease Inhibitor Cocktail Set III (Merck Millipore, #535140). The cell lysates were quantified using a Pierce BCA Protein Assay Kit (Thermo Scientific, #23227). Quantified cell lysates were separated by sodium dodecyl sulfate polyacrylamide gel electrophoresis (SDS-PAGE) and transferred to polyvinylidene difluoride (PVDF) membranes (Merck Millipore, #IPVH00010). The transferred blots were blocked with 5% non-fat skim milk and incubated with antibodies against α-SMA (Sigma-Aldrich, #A5228), fibronectin (Abcam, #ab2413), Myc (Abcam, #ab32072), or α-tubulin (Santa Cruz, #sc-8035) for 12–16 hours at 4°C. After blots were washed with Tris-buffered saline containing 0.1% Tween 20 (TBS-T), they were incubated with secondary antibodies conjugated to horseradish peroxidase (HRP) (Thermo Scientific, #31430) and then washed again. Signals were developed with a chemiluminescence solution (Dogen, #DG-WP100).

#### Fractionation of cell lysates

MyoFBs were treated with purified recombinant proteins (20 nM) for 12 hrs. They were then harvested with trypsin treatment and vigorously washed with PBS. Cytosolic and nuclear fractions were obtained by using NE-PER nuclear and cytoplasmic extraction reagents (Thermo Fisher, #78833) according to the manufacturer’s instruction.

### RNA extraction and quantitative RT-PCR analysis

mRNA expression level was determined by real-time PCR using a QuantiTect SYBR Green real-time PCR Kit (Qiagen, #204243). Total RNA was isolated from MyoFBs with Trizol reagent (Invitrogen, #15596) according to the manufacturer’s instructions. Reverse transcription was performed at 50°C for 20 min, and cDNA was amplified in 20 μL reaction volumes using 10 pmol of primers for 37 cycles: 94°C for 10 s, 57°C for 15 s, and 72°C for 5 s. 18S rRNA was used as an internal control to calculate the relative abundance of the mRNAs. The primer sequences used in this study were as follows: for *α-SMA*, 5’-CCT CAC AGA GAG AGG CTA TTC CT-3’ (Forward) and 5’- GCA GCT CAT AGC TCT CCA G-3’(Reverse); for *Fibronectin*, 5’-CTG GCC GAA AAT ACA TTG TAA A -3’ (Forward) and 5’-CCA CAG TCG GGT CAG GAG-3’(Reverse); for *Vimentin*, 5’-GAG AAC TTT GCC GTT GAA GC-3’ (Forward) and 5’-CGT GAT GCT GAG AAG TTT CG-3’(Reverse); for *CCN2*, 5’-CTT GCG AAG CTG ACC TGG AAG A-3’ (Forward) and 5’-CCG TCG GTA CAT ACT CCA CAG A-3’(Reverse); for *Twist1*, 5’-CAG ACG CAG CGG GTC ATG- 3’ (Forward) and 5’-AGG GCA GCG TGG GGA TGA-3’(Reverse); for *Slug*, 5’-GAC TAC CGC TGC TCC ATT- 3’ (Forward) and 5’-GAG GAG GTG TCA GAT GGA-3’(Reverse); for *Klf4*, 5’-GCA GCC ACC TGG CGA GTC TG-3’ (Forward) and 5’-CCG CCA GCG GTT ATT CGG GG-3’(Reverse); for *18s rRNA*, 5′-GTG GAG CGA TTT GTC TGG TT-3′ (Forward) and 5′-CGC TGA GCC AGT CAG TGT AG-3′ (Reverse).

### Statistical analysis

N numbers represent independent biological observations. Student’s t-test and One-Way Analysis of Variance were used for statistical analyses to determine the significance of the data. An asterisk (*, *p* < 0.05) or a double asterisk (**, *p* < 0.01) indicate significant probability. Data represent the mean ± standard deviation.

## Results

### CCN5 is internalized mainly via clathrin-mediated endocytosis

**Fig 1:**
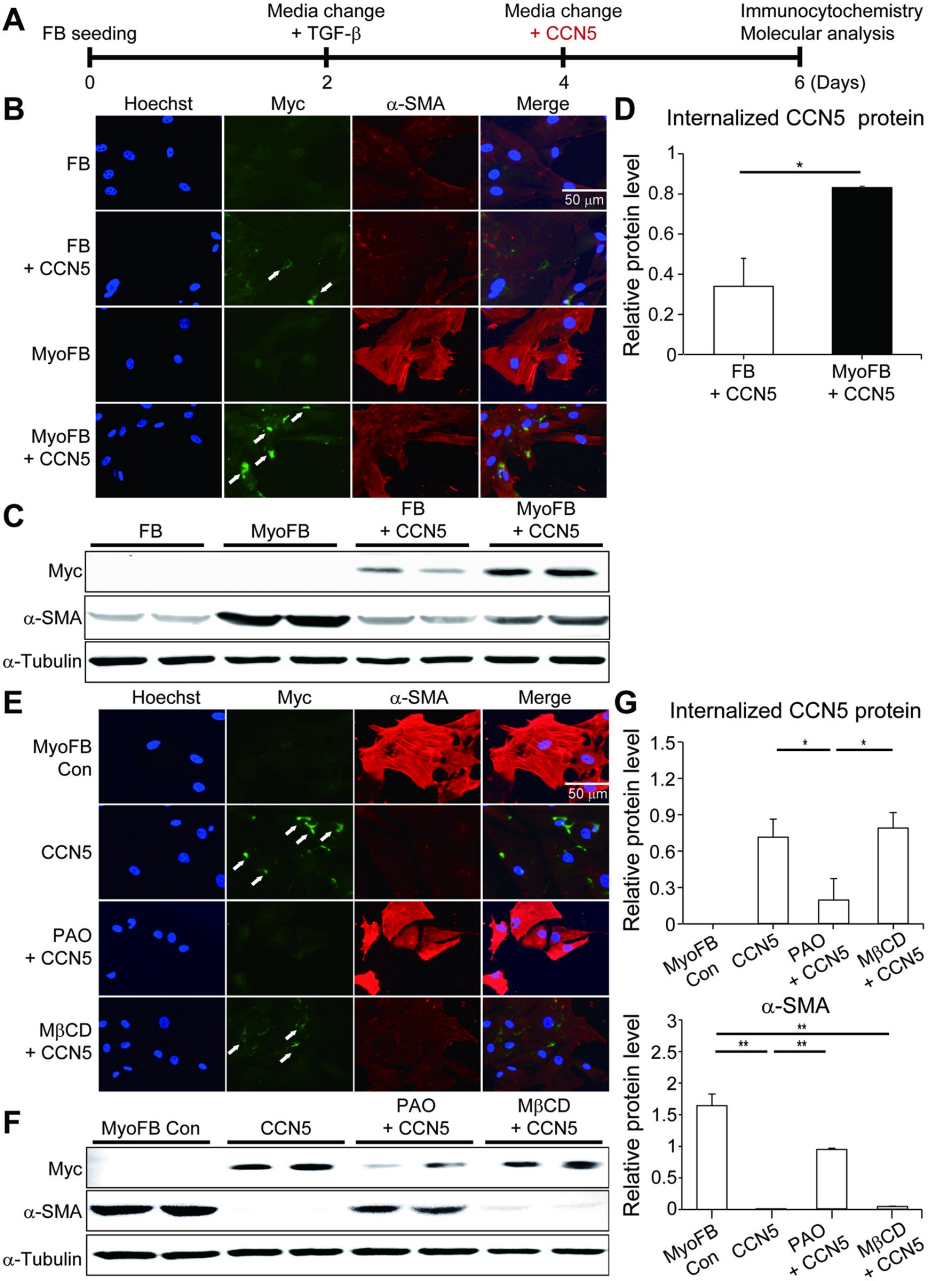
CCN5 is internalized mainly via clathrin-mediated endocytosis. (A) The experimental scheme. FBs were trans-differentiated to MyoFBs by treatment with TGF-β (10 ng/mL). FBs and MyoFBs were treated with purified CCN5 protein (20 nM) for 2 days and then immunostained with anti-Myc (CCN5) and -α-SMA antibodies. Nuclei were stained with Hoechst dye. (B) Representative images are shown. Weak to strong immunogenicity of CCN5 was observed in the majority of the cells. White arrows indicate strong CCN5 signals in cells. (C) Whole cell lysates (30 μg) obtained from FBs and MyoFBs were immunoblotted with antibodies against Myc (CCN5), α-SMA, or α-tubulin. (D) Protein bands on western blots were scanned and plotted. More CCN5 protein was internalized in MyoFBs than in FBs. (E) MyoFBs were treated with CCN5 (20 nM) in the absence or presence of PAO (60 nM) or MβCD (1 mM). Internalization of CCN5 was prevented completely by PAO and partially by MβCD. (F) Whole cell lysates (30 μg) obtained from MyoFBs were immunoblotted with antibodies against Myc (CCN5), α-SMA, or α-tubulin. (G) Protein bands on western blots were scanned and plotted. Scale bar: 50 μm. n = 4.

To investigate the role of CCN5 in the reversal of trans-differentiation of MyoFBs to FBs, we initially induced trans-differentiation of human ventricular FBs by exposure to TGF-β for two days, followed by treatment with purified human CCN5 protein (20 nM) for additional two days (Fig 1A). The recombinant CCN5 protein used in this study has Myc and His tags at the carboxy terminus. The trans-differentiated MyoFBs showed elevated expression of α-smooth muscle actin (α-SMA), a marker for fibrosis. Consistent with our previous reports, immunocytochemistry exhibited that CCN5 significantly reduced the expression of α-SMA in MyoFBs. In parallel, CCN5 was internalized into FBs and MyoFBs, as indicated by intracellular staining for Myc (arrows in Fig 1B). No Myc staining was observed when cells were not permeabilized prior to treatment with antibody (S1 Fig), supporting the validity of our immunostaining experiments. Cells were scraped after rigorous washing and subjected to western blotting, which confirmed that α-SMA expression was significantly reduced in parallel with the cellular uptake of CCN5 (Fig 1C). The CCN5 (Myc) levels on western blots were scanned and plotted (Fig 1D), which revealed that significantly more CCN5 proteins were internalized to MyoFBs than FBs. The molecular basis for this difference was unclear. We intriguingly found that the cellular uptake of CCN5 to FBs was significantly enhanced when culture plates were pre-coated with fibronectin, an extracellular matrix (ECM) protein (S2 Fig). This finding suggests that ECM proteins heavily secreted from MyoFBs are associated with the enhanced cellular uptake of CCN5.

We then evaluated the effects of phenylarsine oxide (PAO), an inhibitor of clathrin-mediated endocytosis, and methyl-β-cyclodextrin (MβCD), an inhibitor of lipid raft-mediated endocytosis, on the cellular uptake of CCN5. Immunocytochemistry showed that PAO significantly inhibited the internalization of CCN5. Notably, CCN5 failed to reduce expression of α-SMA in the presence of PAO (Fig 1E). These findings were confirmed by western blotting followed by quantitation of protein bands, in which PAO inhibited the cellular uptake of CCN5 with no reduced expression of α-SMA (Fig 1F and 1G). Chlorpromazine, another inhibitor of clathrin-mediated endocytosis, exhibited similar effects on the cellular uptake of CCN5 (S3 Fig). MβCD was significantly less efficient compared to PAO in inhibiting the cellular uptake of CCN5 and expression of α-SMA (Fig 1E–1G).

Collectively, these data indicate that CCN5 is internalized mainly via clathrin-mediated endocytosis, and that this internalization is critical for the function of CCN5 in reversing the phenotype of MyoFBs.

### The TSP-1 domain of CCN5 is essential for its internalization and nuclear localization

**Fig 2:**
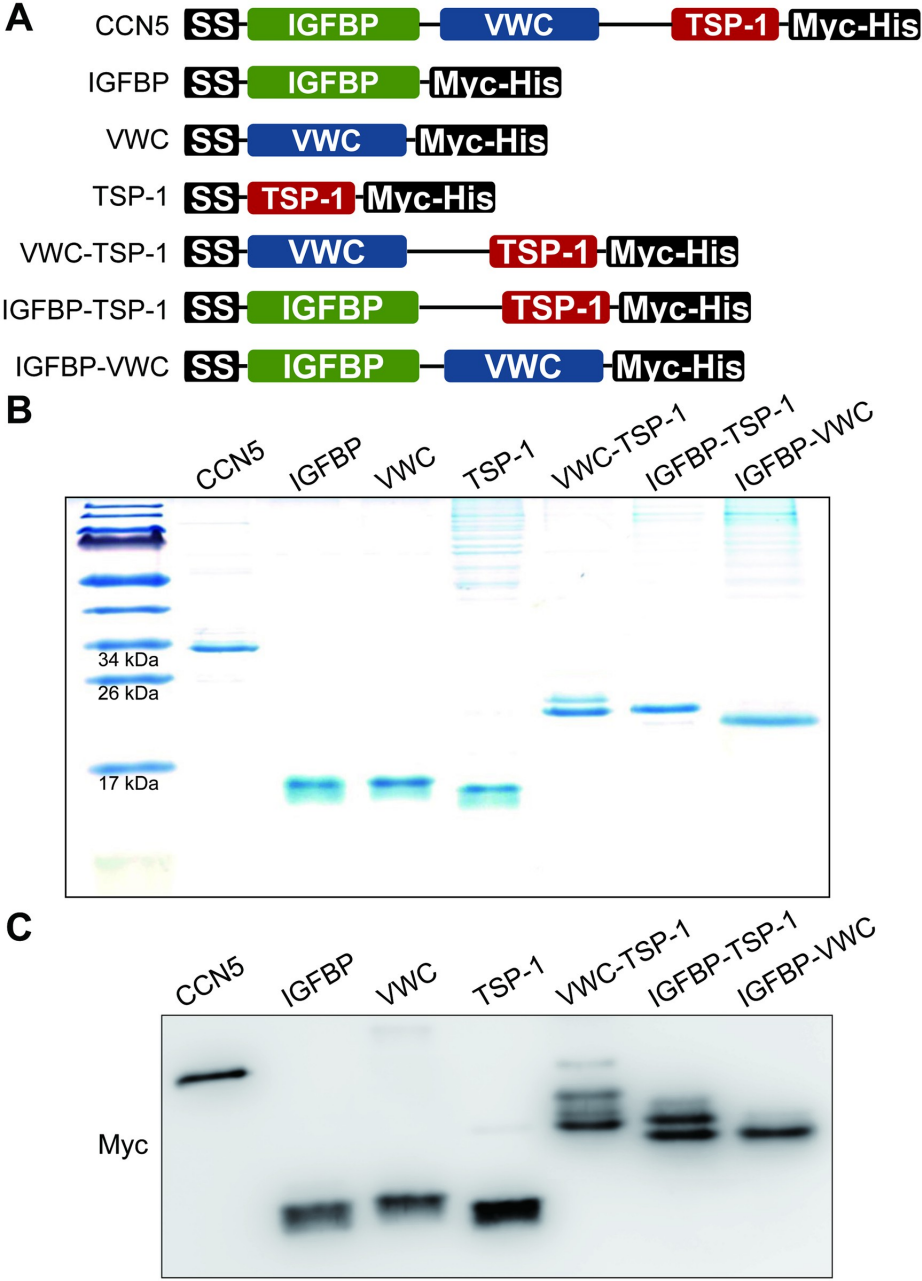
Constructs for the single and paired-domain proteins of CCN5. (A) Constructs for full-length CCN5 and single domain and paired-domain proteins of CCN5 are shown. The constructs were expressed in HEK 293-F cells and the corresponding proteins were purified from culture media using the His tag. The purified proteins (1 μg) were subjected to SDS-PAGE and stained with Coomassie blue (B). The proteins (200 ng) were also subjected to western blotting with anti-Myc antibody (C).

To dissect the function of each CCN5 domain during endocytosis, we generated recombinant constructs that encode a single domain only (Fig 2A; IGFBP, VWC, and TSP-1) or pairs of domains (Fig 2A; VWC-TSP-1, IGFBP-TSP-1, and IGFBP-VWC). These recombinant proteins were tagged with Myc and His at their carboxy termini. The amino acid sequences of the recombinant proteins are shown in S4 Fig. The recombinant proteins were expressed in HEK 293-F cells and purified to near homogeneity using the His tags as shown by Coomassie staining of SDS-PAGE gels (Fig 2B). The purified proteins were loaded onto SDS-PAGE gels and probed with anti-Myc antibody to confirm the identity of the purified proteins (Fig 2C).

**Fig 3:**
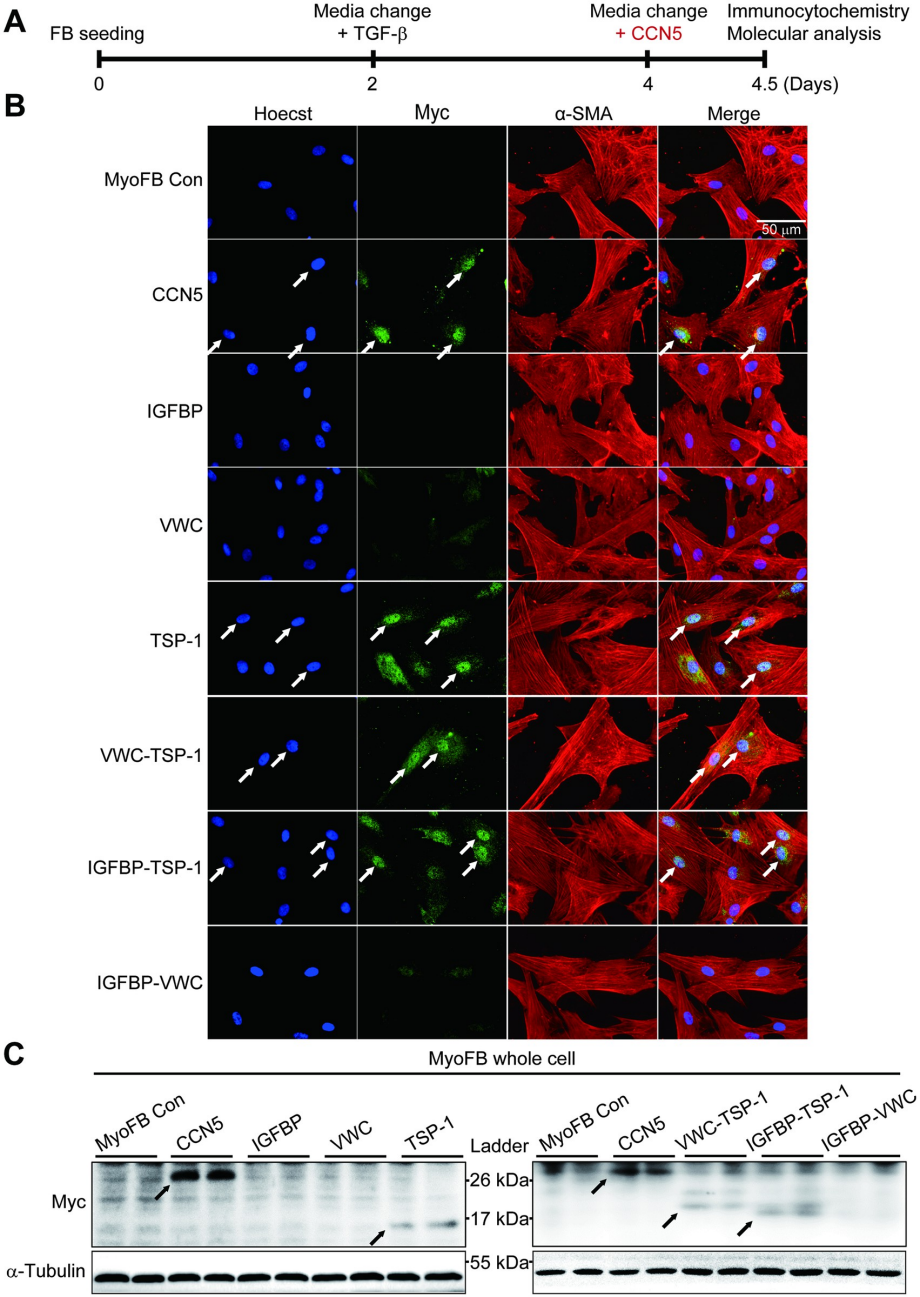
The TSP-1 domain is essential for internalization and nuclear localization of CCN5. (A) The experimental scheme is shown. FBs were trans-differentiated to MyoFBs by treatment with TGF-β (10 ng/mL). MyoFBs were then treated with purified CCN5 protein, or with single or paired domains of CCN5 (20 nM) for 12 hours, and then immunostained for Myc (CCN5) and α-SMA. Nuclei were stained with Hoechst dye. (B) Representative images are shown. White arrows indicate CCN5 protein localized in the nucleus. (C) Whole cell lysates were immunoblotted with anti-Myc (CCN5) and anti-α-tubulin antibodies. (D, E) MyoFBs treated with CCN5 and single domain proteins (D) or paired-domain proteins (E) were fractionated into cytosolic and nuclear fractions. Western blotting was performed with anti-Myc antibody to detect CCN5 and other recombinant proteins. α-tubulin and histone H3 were served as markers for cytosolic and nuclear fractions, respectively. Scale bar: 50 μm.

MyoFBs were treated with 20 nM of each purified recombinant protein. Intracellular localization of CCN5 was first observed at 3 hours and reached a peak at 12–16 hours post-treatment, as observed by immunostaining with an anti-Myc antibody (S5 Fig). Thus, localization of the recombinant proteins was examined at 12 hours after treatment (Fig 3A). It is of note that prominent α-SMA staining remained at this time point in all treated groups. Among the single domain proteins, only TSP-1 was localized intracellularly, including in the nucleus. Among the paired-domain proteins, VWC-TSP-1 and IGFBP-TSP-1, but not IGFBP-VWC, were localized intracellularly (arrows in Fig 3B). Prominent nuclear localization of all of these recombinant proteins was observed. Confocal microscopy further confirmed the nuclear localization of the internalized recombinant proteins (S6 Fig). Western blotting confirmed that TSP-1, VWC-TSP-1, and IGFBP-TSP-1, as well as full-length CCN5, were present within cells (arrows in Fig 3C). MyoFBs treated with recombinant proteins were lysed and fractionated into cytosolic and nuclear fractions, which were further analyzed by western blotting. Full-length CCN5 was present in both cytosolic and nuclear fractions. Among the single domain proteins, only TSP-1 was found in cytosolic and nuclear fractions (Fig 3D). All the paired-domain proteins were found in cytosolic fraction, and VWC-TSP-1 and IGFBP-TSP-1, but not IGFBP-VWC, were present in nuclear fraction (Fig 3E).

These data indicate that TSP-1 is necessary and sufficient for internalization and nuclear localization of CCN5.

### The TSP-1 domain is necessary but not sufficient for the function of CCN5

**Fig 4:**
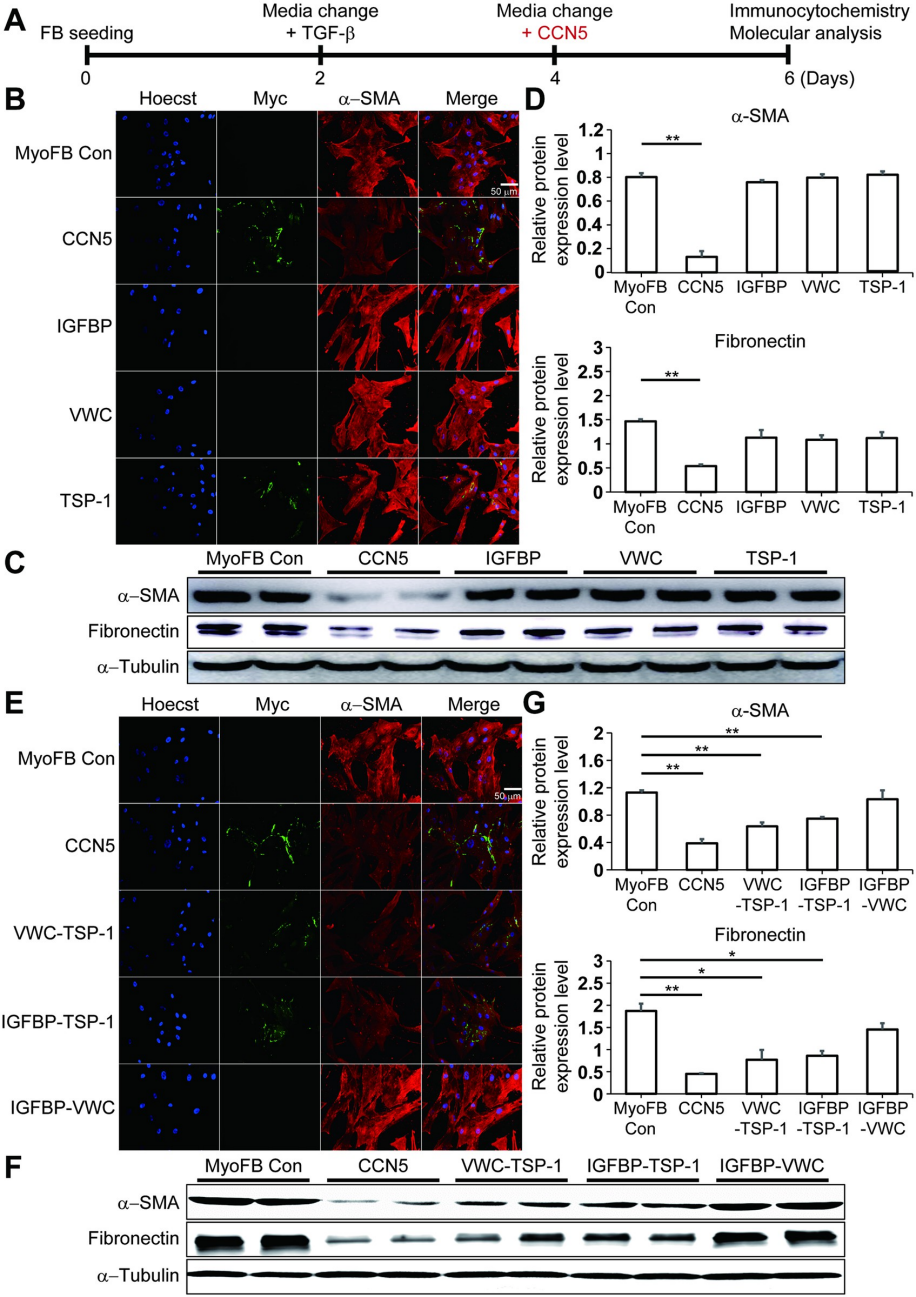
The TSP-1 domain is necessary but not sufficient for the function of CCN5. (A) The experimental scheme is shown. FBs were trans-differentiated to MyoFBs by treatment with TGF-β (10 ng/mL). MyoFBs were then treated for 2 days with purified CCN5 protein or with single domain or paired domains of CCN5 (20 nM). Cells were immunostained for Myc (CCN5), α-SMA, and nuclei were stained with Hoechst dye. (B, E) Representative images are shown. (C, F) Whole cell lysates (30 μg) obtained from MyoFBs were immunoblotted with anti-α-SMA, -fibronectin, and -α-tubulin antibodies. (D, G) Protein bands on western blots were scanned and plotted. Scale bar: 50 μm. n = 6.

We further examined the ability of the recombinant proteins to reverse the phenotype of MyoFBs at two days post-treatment (Fig 4A). As observed with immunocytochemistry, none of the single domain proteins was able to reduce the elevated expression of α-SMA (Fig 4B). The levels of α-SMA and fibronectin were quantitated by western blotting. The data also showed that full-length CCN5, but none of the single domain proteins, reduced the expression of α-SMA and fibronectin significantly (Fig 4C and 4D). Among the paired-domain proteins, VWC-TSP-1 and IGFBP-TSP-1, but not IGFBP-VWC, reduced the expression of α-SMA significantly (Fig 4E). These findings were also confirmed by western blotting (Fig 4F and 4G).

These data indicate that the TSP-1 domain is necessary but not sufficient for the function of CCN5, at least in the context of reversing trans-differentiation of MyoFBs to FBs.

## Discussion

CCN family proteins are supposed to be secreted along with their amino-terminal signal peptides [17–21]. However, these proteins are detected in intracellular as well as extracellular spaces [14, 15, 22–27]. In particular, CCN5 localizes in the cytosol and nucleus in diverse cells as determined by immunocytochemistry and cellular fractionation experiments [16, 17]. In this study, we showed that exogenously-added CCN5 proteins are internalized into cytosol mainly via clathrin-mediated endocytosis, followed by transportation to the nucleus of cardiac FBs and MyoFBs (Fig 1). Furthermore, we showed that internalization of CCN5 was critical for inducing the reverse trans-differentiation of MyoFBs to FBs (Fig 4). Notably, a previous study showed that CCN5 functions as a transcriptional repressor during regulation of TGF-β receptor II expression in human tumor breast tissues [16]. Therefore, we suggest that CCN5 may evoke a gene expression program that induces the reverse trans-differentiation of cardiac MyoFBs. Quantitative RT-PCR analyses revealed that CCN5 reduced the transcriptional level of fibrosis markers, α-SMA, fibronectin, and vimentin (S7A Fig). In consistent with our previous results [10, 11], CCN5 down-regulated CCN2, a potent pro-fibrotic molecule, at the transcriptional level (S7B Fig). Intriguingly, CCN5 altered the expression levels of transcriptional co-factors involved in cell differentiation including Twist1, Slug, and Klf4 (S7C Fig). Further studies are warranted to elucidate the signaling cascade evoked by CCN5 during the reverse trans-differentiation of cardiac MyoFBs.

Although CCN family proteins are generally considered to be secreted to and function at cell surface or ECM, they were often localized to the nucleus of specific cell types. For example, CCN1 was localized in the nucleus of mechanically stretched bladder smooth muscle cells [28]. In human mesangial cells, CCN2 was shown to be internalized from cell surface to endosomes and accumulated in juxtanuclear organelle. CCN2 were then translocated to the nucleus, where it might affect transcriptional activity [29]. It is unclear how the nuclear translocation of CCN2 is regulated. The carboxy-terminal (CT) domain of CCN3 appeared to contain a potential nuclear localization sequence (NLS). Despite the presence of this sequence, CCN3 is normally secreted to ECM presumably because the amino-terminal signal sequence overrides the NLS activity of the sequence [30]. Amino terminus-truncated CCN3 variants, identified from Hela and other tumor cells, are localized in the nucleus, which is presumably associated with oncogenicity of the CCN3 variants [31]. Therefore, it seems that multiple mechanisms are involved in the nuclear localization of CCN family proteins.

TSP-1, an adhesive glycoprotein with a molecular weight of ~450 kDa, is a member of the thrombospondin family of proteins that bind to fibrinogen, fibronectin, laminin, collagen, and integrin. TSP-1 is endocytosed after interacting with cell surface receptors such as low-density lipoprotein-related protein (LRP-1) [32–34]. TSP-1 contains three thrombospondin type 1 repeats (TSR), which are essential for its function [35, 36]. The role of TSR has not been associated with endocytosis nor nuclear localization. The TSP-1 domain of CCN5 contains one TSR-like sequence. Our data suggest that the TSP-1 domain must contain signals for both endocytosis and nuclear localization. Internalization of CCN5 was prominently enhanced when the culture plates were pre-coated with fibronectin (S2 Fig). In parallel, CCN5 exhibited a strong affinity to fibronectin. TSP-1, VWC-TSP-1, and IGFBP-TSP-1 also bind to fibronectin with a strong affinity (Unpublished). These data implies that the TSP-1 domain plays a critical role for the internalization of CCN5 through binding to fibronectin.

In conclusion, we showed that the TSP-1 domain of CCN5 plays an essential role in the reversal of trans-differentiation of MyoFBs to FBs.

## Supporting information

S1 Fig Immunostaining images of MyoFBs with or without permeabilization. MyoFBs were treated with CCN5 protein (20 nM) for 12 hours. Cells were either permeabilized with 0.5% Triton X-100 or non-permeabilized, and then anti-Myc antibody was treated to detect CCN5. Note that Myc (CCN5) signal was observed only when cells were permeabilized. Scale bar: 100 μm.
(JPG)

S2 Fig ECM-dependent CCN5 internalization in FBs. (A) The experimental scheme is shown. Culture plates were pre-coated with ECM molecules (0.2% gelatin, 5 μg/cm^2^ fibronectin, 5 μg/cm^2^ collagen, 2 μg/cm^2^ laminin) for 30 mins at RT. FBs were then seeded onto these pre-coated plates. CCN5 protein (20 nM) was treated for two days. (B) Representative images are shown. FBs were immunostained for Myc (CCN5). Nuclei were stained with Hoechst dye. Notably, more CCN5 protein was internalized in the presence of fibronectin. (C) Whole cells lysates (30 μg) were immunoblotted with anti-Myc (CCN5) and -α-tubulin antibodies. (D) Protein bands on western blots were scanned and plotted. Scale bar: 20 μm. n = 4. *p<0.05, **p<0.01.
(JPG)

S3 Fig Two different kinds of clathrin-mediated endocytosis inhibition assay. (A) The experimental scheme is shown. FBs were trans-differentiated to MyoFBs by treatment with TGF-α (10 ng/mL) for 2 days. MyoFBs were pre-incubated with 60 nM phenylarsine oxide (PAO) or 10 ug/mL chlorpromazine for 30 mins at 37°C. CCN5 protein (20 nM) was then added for 2 days. (B) Representative images are shown. MyoFBs were immunostained for Myc (CCN5). Nuclei were stained with Hoechst dye. White arrows indicate the internalized CCN5 protein. Both PAO and chlorpromazine significantly inhibited the cellular uptake of CCN5. (C) Whole cells lysates (30 μg) were immunoblotted with anti-Myc (CCN5), -α-SMA and -α-tubulin antibodies. (D) Protein bands on western blots were scanned and plotted. Scale bar: 50 μm. n = 4. *p<0.05, **p<0.01.
(JPG)

S4 Fig Amino acid sequences of full-length CCN5, single domain and paired-domain CCN5 proteins. IGFBP, VWC, TSP-1 domains are shown in green, blue, and red, respectively.
(JPG)

S5 Fig Time-dependent subcellular localization of CCN5. MyoFBs were treated with CCN5 protein (20 nM) for 3, 6, 12, 24, and 48 hours. Cells were permeabilized with 0.5% Triton X-100, and then anti-CCN5 antibody (Genscript, # A01012) was treated to detect CCN5. Nuclei were stained with Hoechst dye. Note that nuclear localization signal of CCN5 was maximally observed at 12 hrs after treatment. Scale bar: 100 μm.
(JPG)

S6 Fig Confocal images of CCN5 proteins nuclear localization. (A) The experimental scheme is shown. FBs were trans-differentiated to MyoFBs by treatment with TGF-β (10 ng/mL) for two days, and then treated with full-length CCN5, TSP-1, VWC-TSP-1, and IGFBP-TSP-1 for 12 hours. (B) Representative confocal images are shown. MyoFBs were immunostained for Myc (CCN5) and α-SMA. Nuclei were stained with Hoechst dye. Anti-rabbit IgG antibody conjugated with Alexa Fluor 488 (Invitrogen, A11008) or anti-mouse IgG antibody conjugated with Alexa Fluor 594 (Invitrogen, A11032) were used as secondary antibodies. Images were obtained with Olympus FV3000RS confocal microscope. White arrows indicate internalized CCN5 protein. Scale bar: 50 μm.
(JPG)

S7 Fig mRNA expression level of fibrosis and EMT marker in CCN5 treated-MyoFBs. Quantitative RT-PCR results of MyoFBs. Cells were treated with CCN5 for 2 days. (A) Transcript levels of fibrosis marker. (B) Transcript level of CCN2. (C) Transcript levels of transcriptional co-factors involved in cell differentiation. 18S rRNA was used as an internal control to calculate the relative abundance of the mRNAs. n = 4. *p<0.05, **p<0.01.
(JPG)

S1 Raw images (PDF)
