# Supplementary figures and images for: The TSP-1 domain of the matricellular protein CCN5 is essential for its nuclear localization and anti-fibrotic function
Source: PLoS One. 2022 Apr 27;17(4):e0267629. doi: 10.1371/journal.pone.0267629 (PMC9045603; doi:10.1371/journal.pone.0267629)

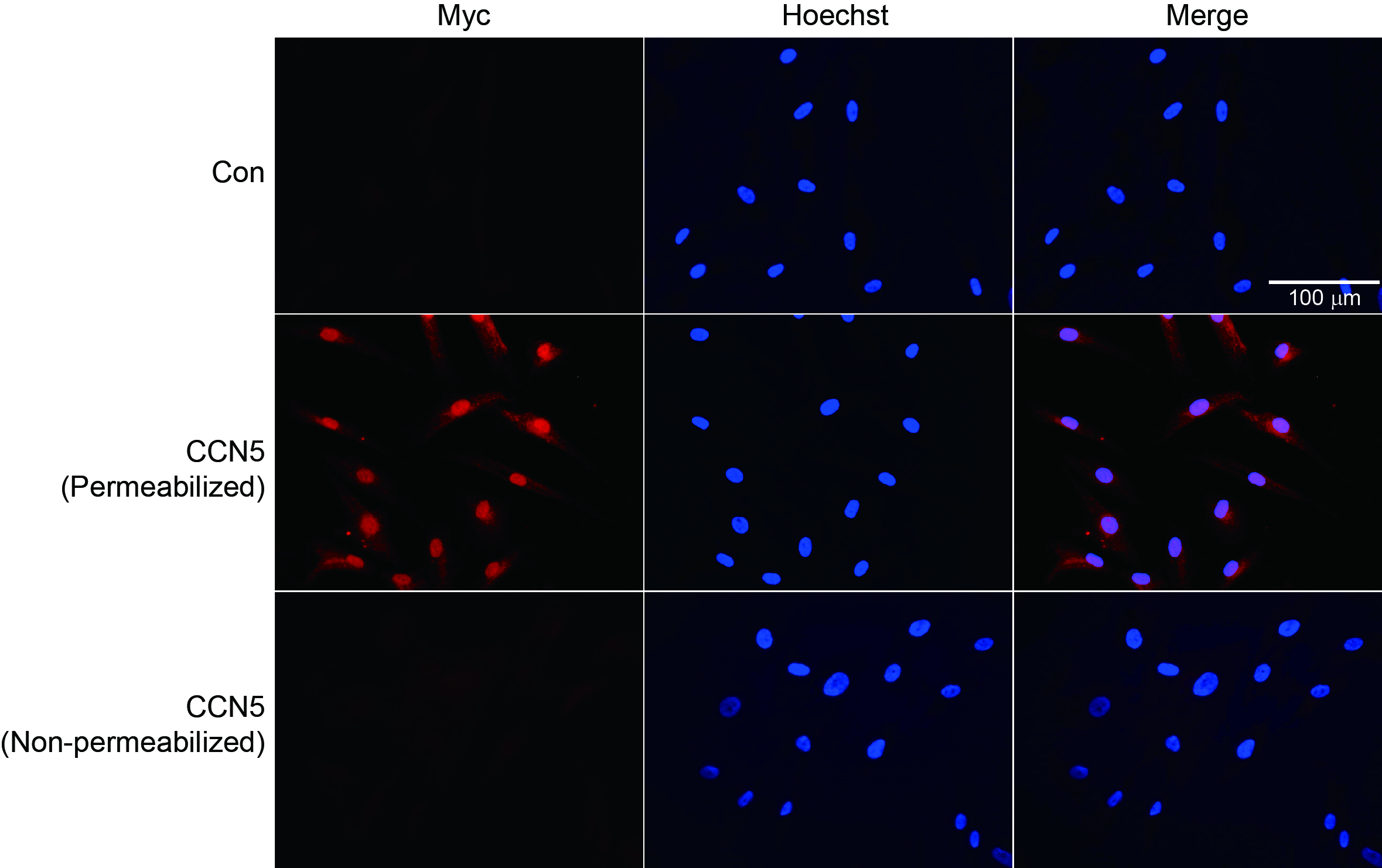

Supplement: S1 Fig — MyoFBs were treated with CCN5 protein (20 nM) for 12 hours. Cells were either permeabilized with 0.5% Triton X-100 or non-permeabilized, and then anti-Myc antibody was treated to detect CCN5. Note that Myc (CCN5) signal was observed only when cells were permeabilized. Scale bar: 100 μm. (JPG) [file pone.0267629.s001.jpg]

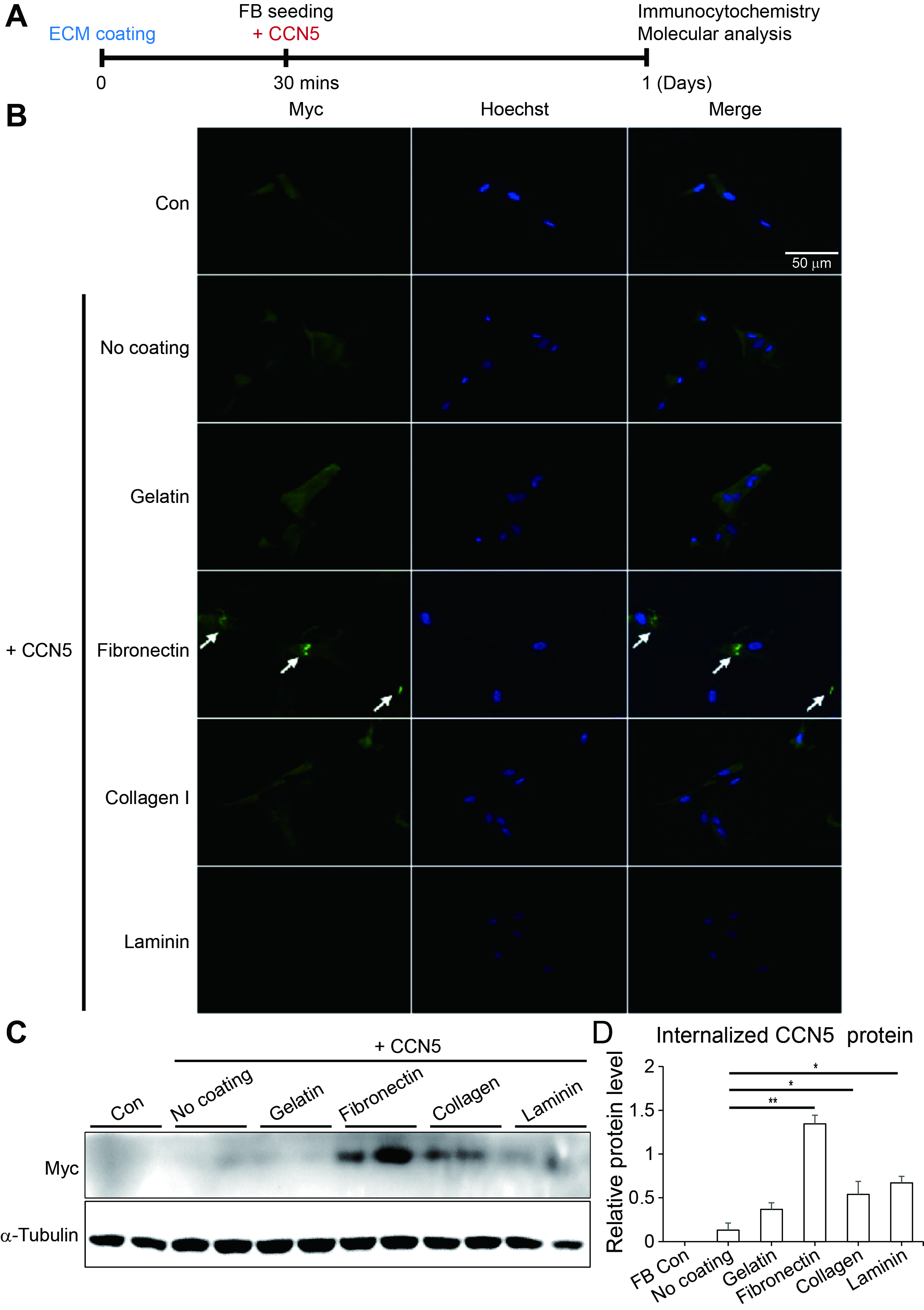

Supplement: S2 Fig — (A) The experimental scheme is shown. Culture plates were pre-coated with ECM molecules (0.2% gelatin, 5 μg/cm2 fibronectin, 5 μg/cm2 collagen, 2 μg/cm2 laminin) for 30 mins at RT. FBs were then seeded onto these pre-coated plates. CCN5 protein (20 nM) was treated for two days. (B) Representative images are shown. FBs were immunostained for Myc (CCN5). Nuclei were stained with Hoechst dye. Notably, more CCN5 protein was internalized in the presence of fibronectin. (C) Whole cells lysates (30 μg) were immunoblotted with anti-Myc (CCN5) and -α-tubulin antibodies. (D) Protein bands on western blots were scanned and plotted. Scale bar: 20 μm. n = 4. *p<0.05, **p<0.01. (JPG) [file pone.0267629.s002.jpg]

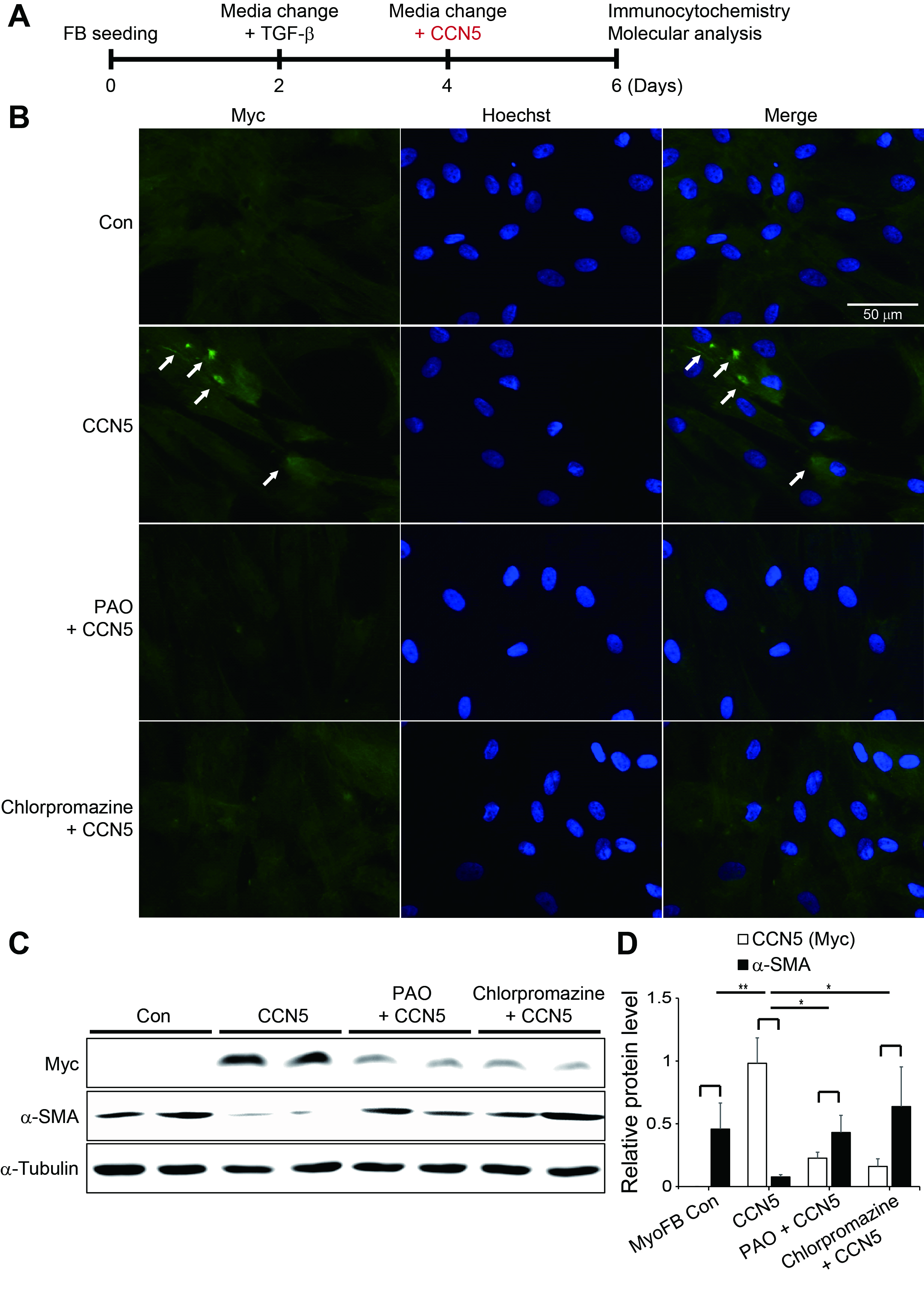

Supplement: S3 Fig — (A) The experimental scheme is shown. FBs were trans-differentiated to MyoFBs by treatment with TGF-α (10 ng/mL) for 2 days. MyoFBs were pre-incubated with 60 nM phenylarsine oxide (PAO) or 10 ug/mL chlorpromazine for 30 mins at 37°C. CCN5 protein (20 nM) was then added for 2 days. (B) Representative images are shown. MyoFBs were immunostained for Myc (CCN5). Nuclei were stained with Hoechst dye. White arrows indicate the internalized CCN5 protein. Both PAO and chlorpromazine significantly inhibited the cellular uptake of CCN5. (C) Whole cells lysates (30 μg) were immunoblotted with anti-Myc (CCN5), -α-SMA and -α-tubulin antibodies. (D) Protein bands on western blots were scanned and plotted. Scale bar: 50 μm. n = 4. *p<0.05, **p<0.01. (JPG) [file pone.0267629.s003.jpg]

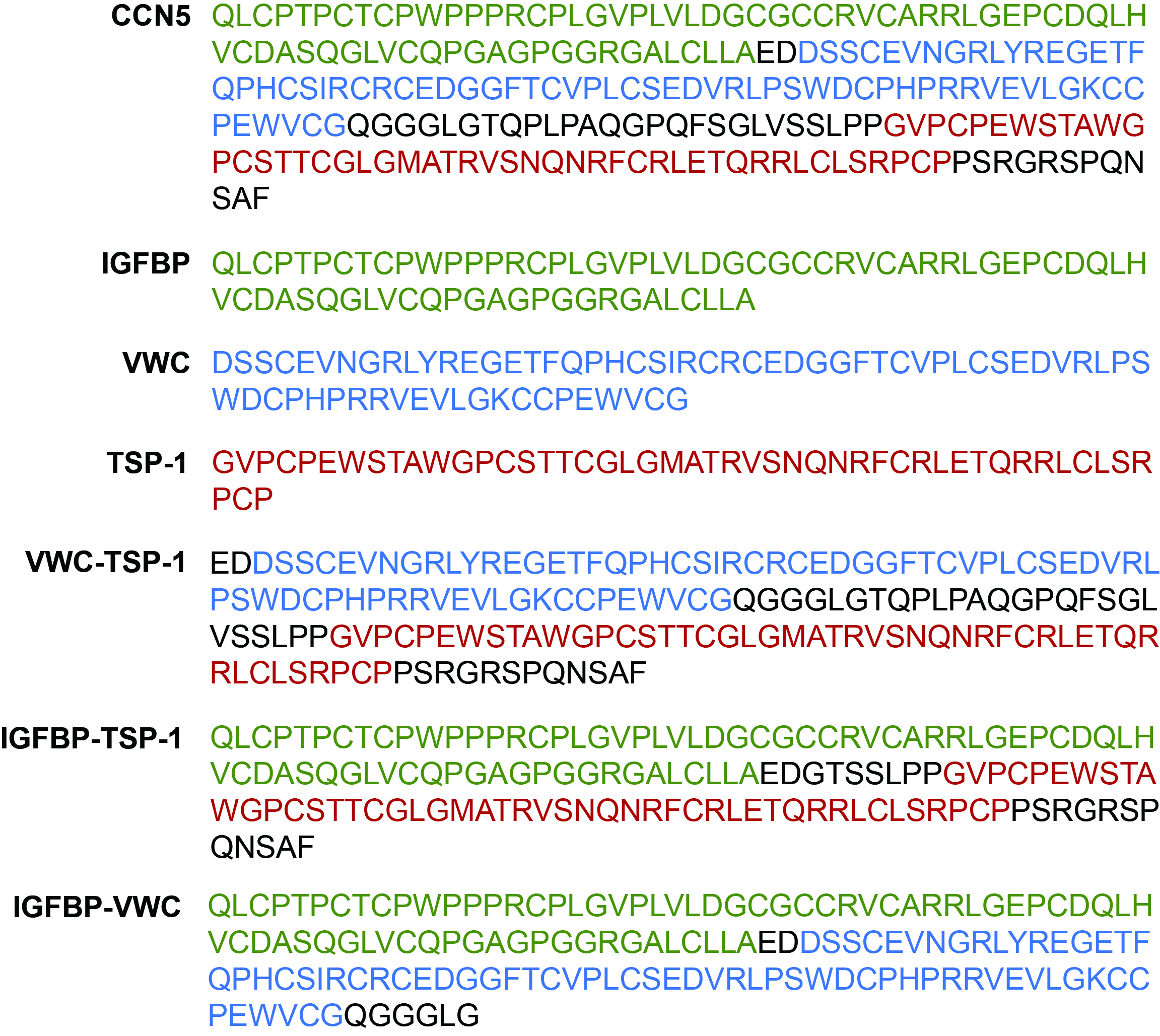

Supplement: S4 Fig — IGFBP, VWC, TSP-1 domains are shown in green, blue, and red, respectively. (JPG) [file pone.0267629.s004.jpg]

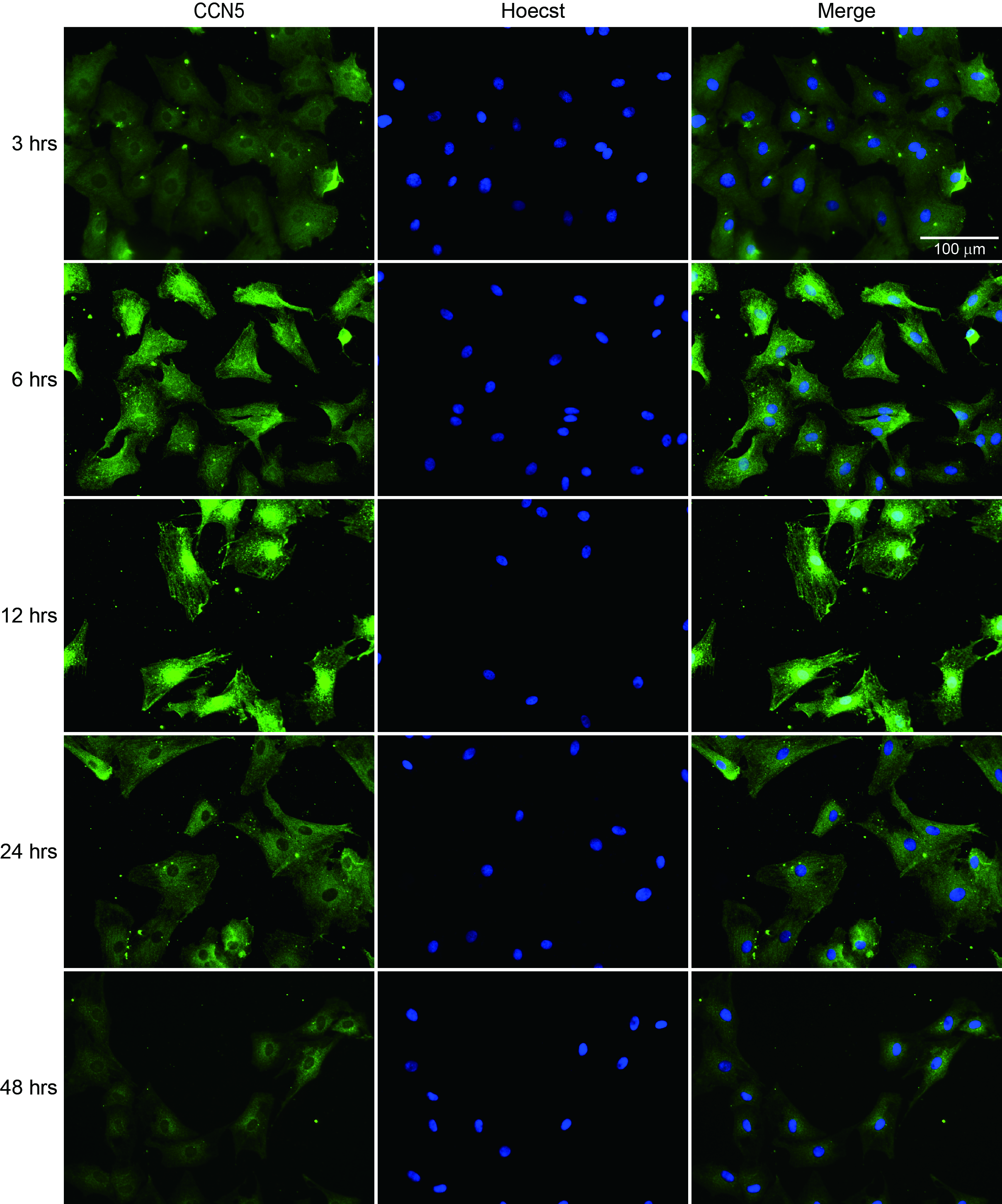

Supplement: S5 Fig — MyoFBs were treated with CCN5 protein (20 nM) for 3, 6, 12, 24, and 48 hours. Cells were permeabilized with 0.5% Triton X-100, and then anti-CCN5 antibody (Genscript, # A01012) was treated to detect CCN5. Nuclei were stained with Hoechst dye. Note that nuclear localization signal of CCN5 was maximally observed at 12 hrs after treatment. Scale bar: 100 μm. (JPG) [file pone.0267629.s005.jpg]

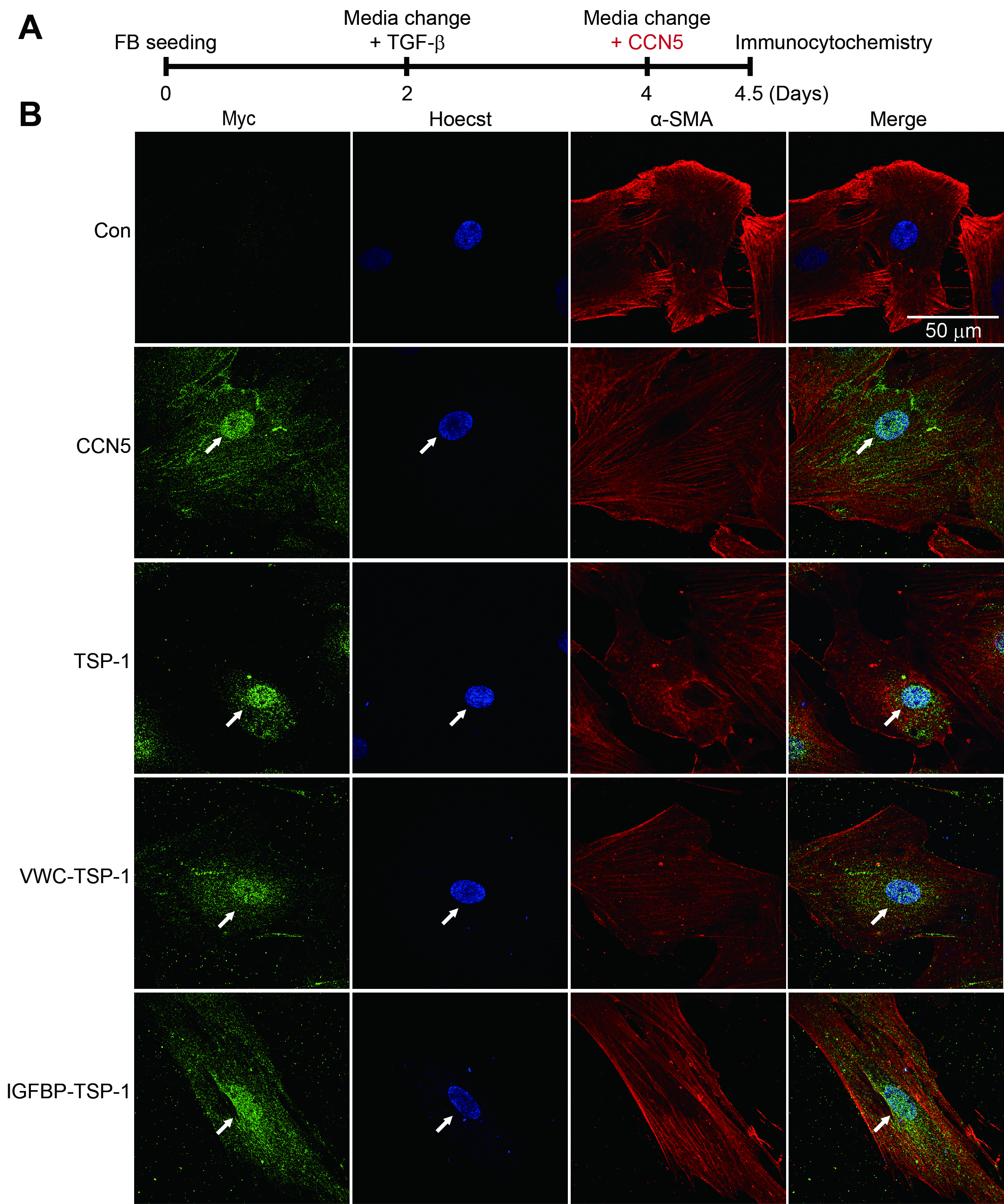

Supplement: S6 Fig — (A) The experimental scheme is shown. FBs were trans-differentiated to MyoFBs by treatment with TGF-β (10 ng/mL) for two days, and then treated with full-length CCN5, TSP-1, VWC-TSP-1, and IGFBP-TSP-1 for 12 hours. (B) Representative confocal images are shown. MyoFBs were immunostained for Myc (CCN5) and α-SMA. Nuclei were stained with Hoechst dye. Anti-rabbit IgG antibody conjugated with Alexa Fluor 488 (Invitrogen, A11008) or anti-mouse IgG antibody conjugated with Alexa Fluor 594 (Invitrogen, A11032) were used as secondary antibodies. Images were obtained with Olympus FV3000RS confocal microscope. White arrows indicate internalized CCN5 protein. Scale bar: 50 μm. (JPG) [file pone.0267629.s006.jpg]

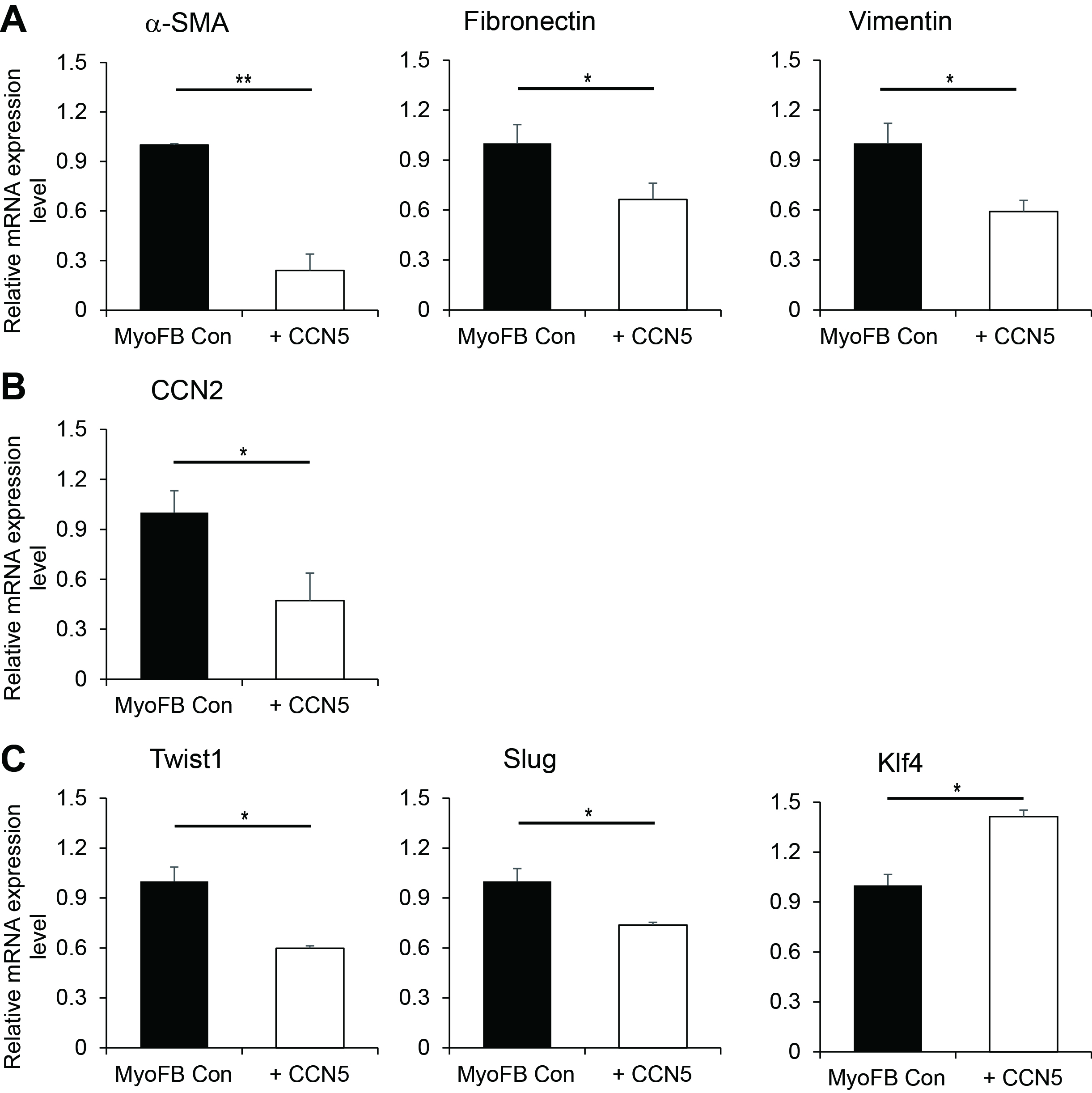

Supplement: S7 Fig — Quantitative RT-PCR results of MyoFBs. Cells were treated with CCN5 for 2 days. (A) Transcript levels of fibrosis marker. (B) Transcript level of CCN2. (C) Transcript levels of transcriptional co-factors involved in cell differentiation. 18S rRNA was used as an internal control to calculate the relative abundance of the mRNAs. n = 4. *p<0.05, **p<0.01. (JPG) [file pone.0267629.s007.jpg]
