# Supplementary material for: The TSP-1 domain of the matricellular protein CCN5 is essential for its nuclear localization and anti-fibrotic function
Source: PLoS One. 2022 Apr 27;17(4):e0267629. doi: 10.1371/journal.pone.0267629 (PMC9045603; doi:10.1371/journal.pone.0267629)

Figure 1-C

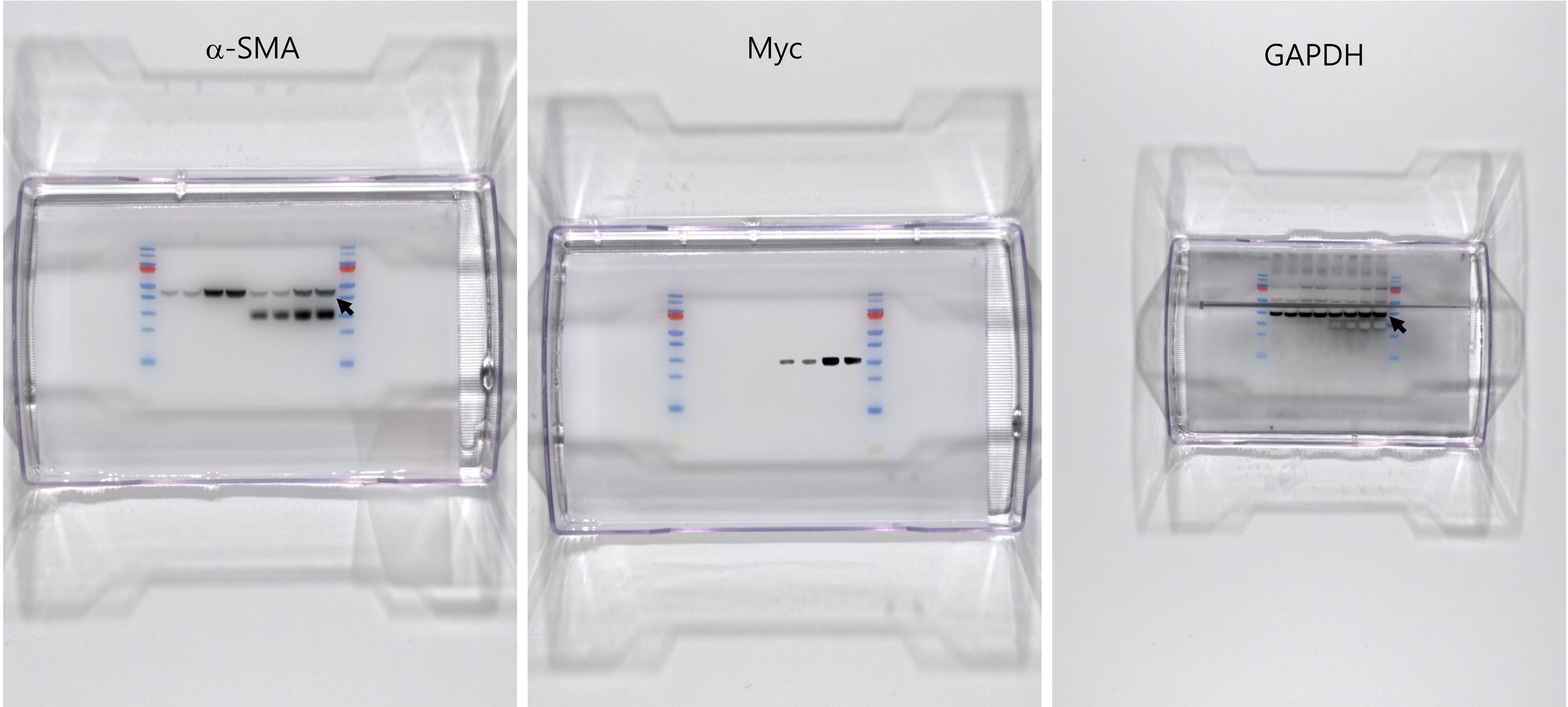

Figure 1-F

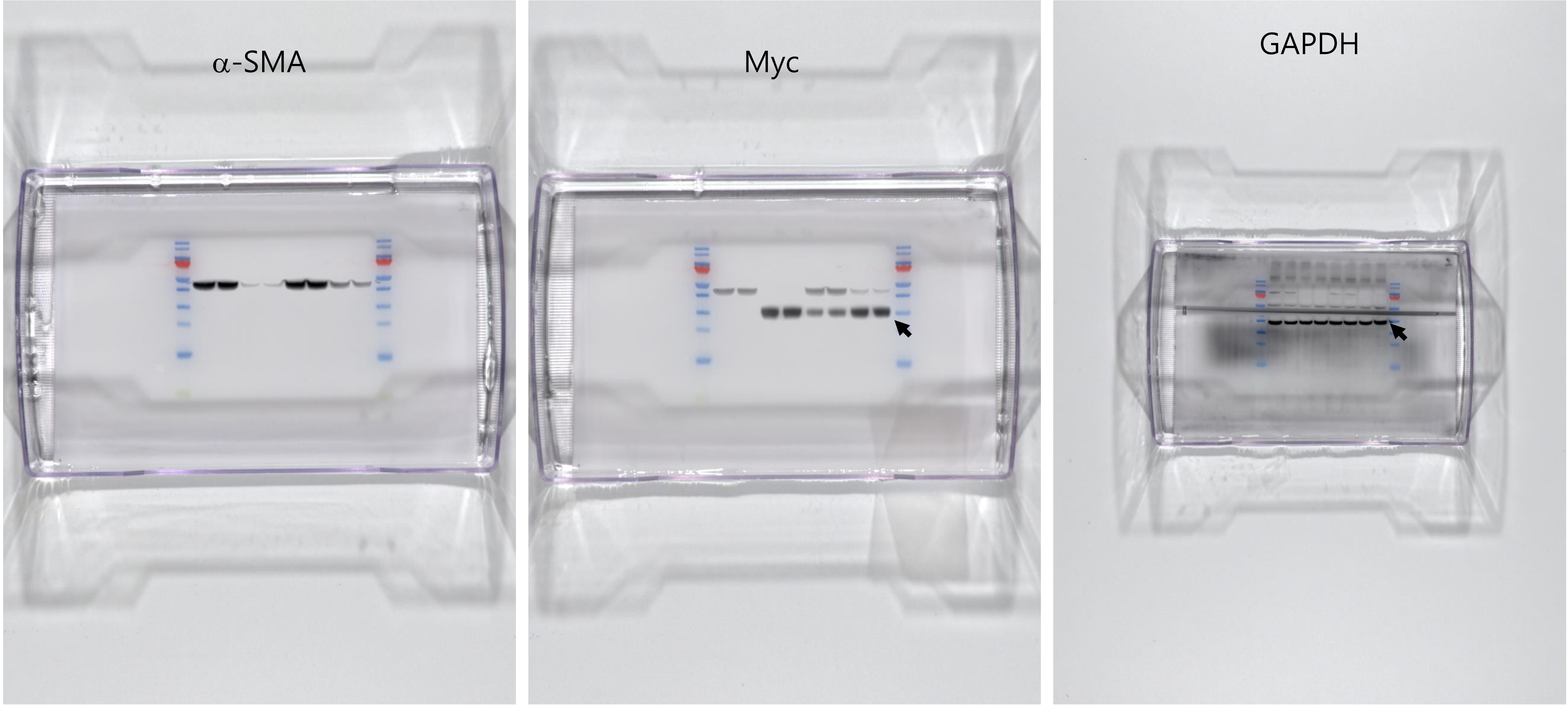

Figure 2-B, C

Coomassie blue staining

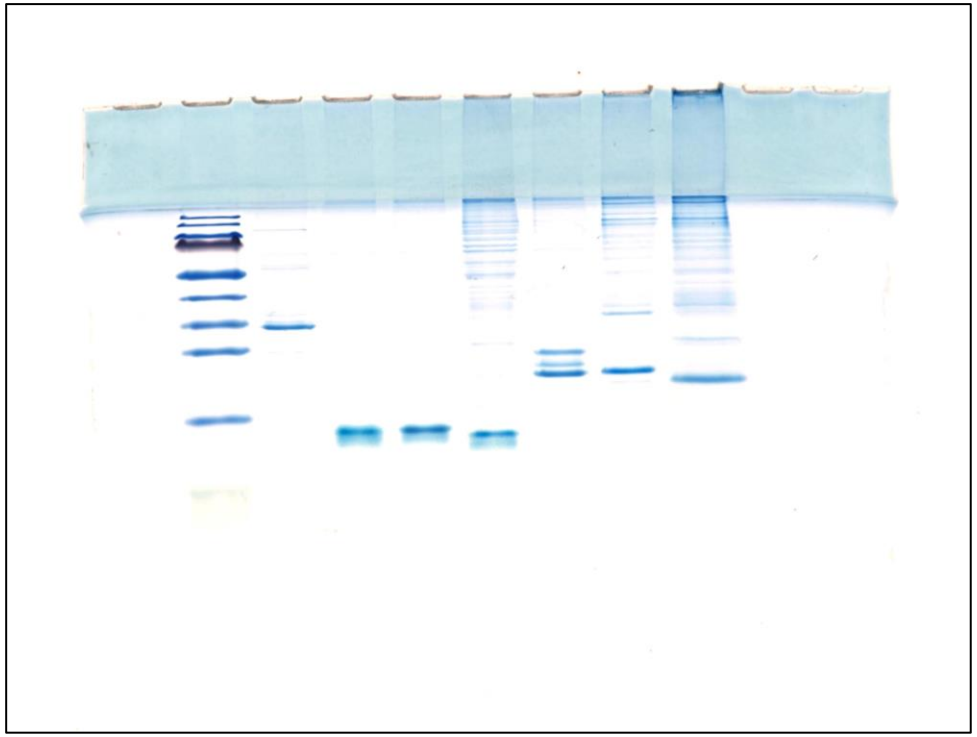

Myc

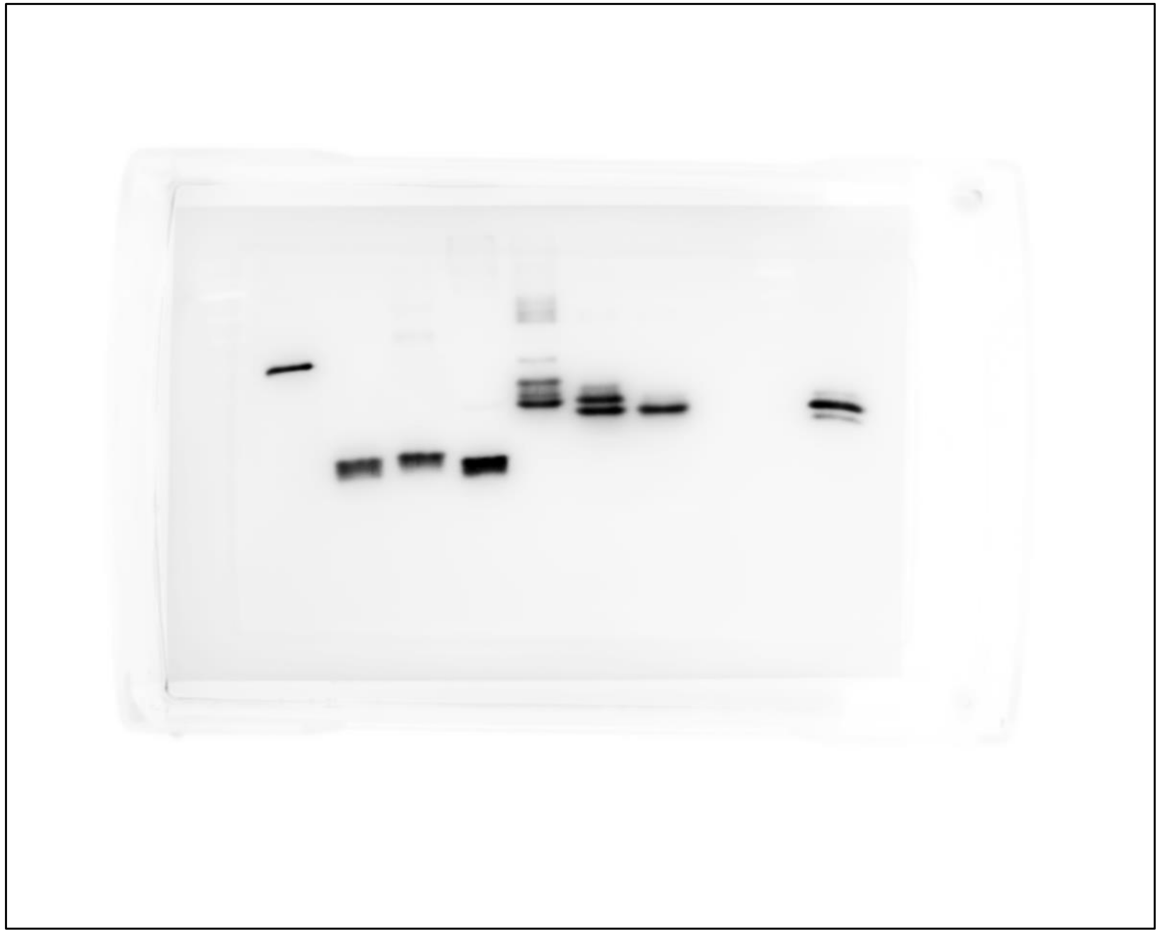

Figure 3-C

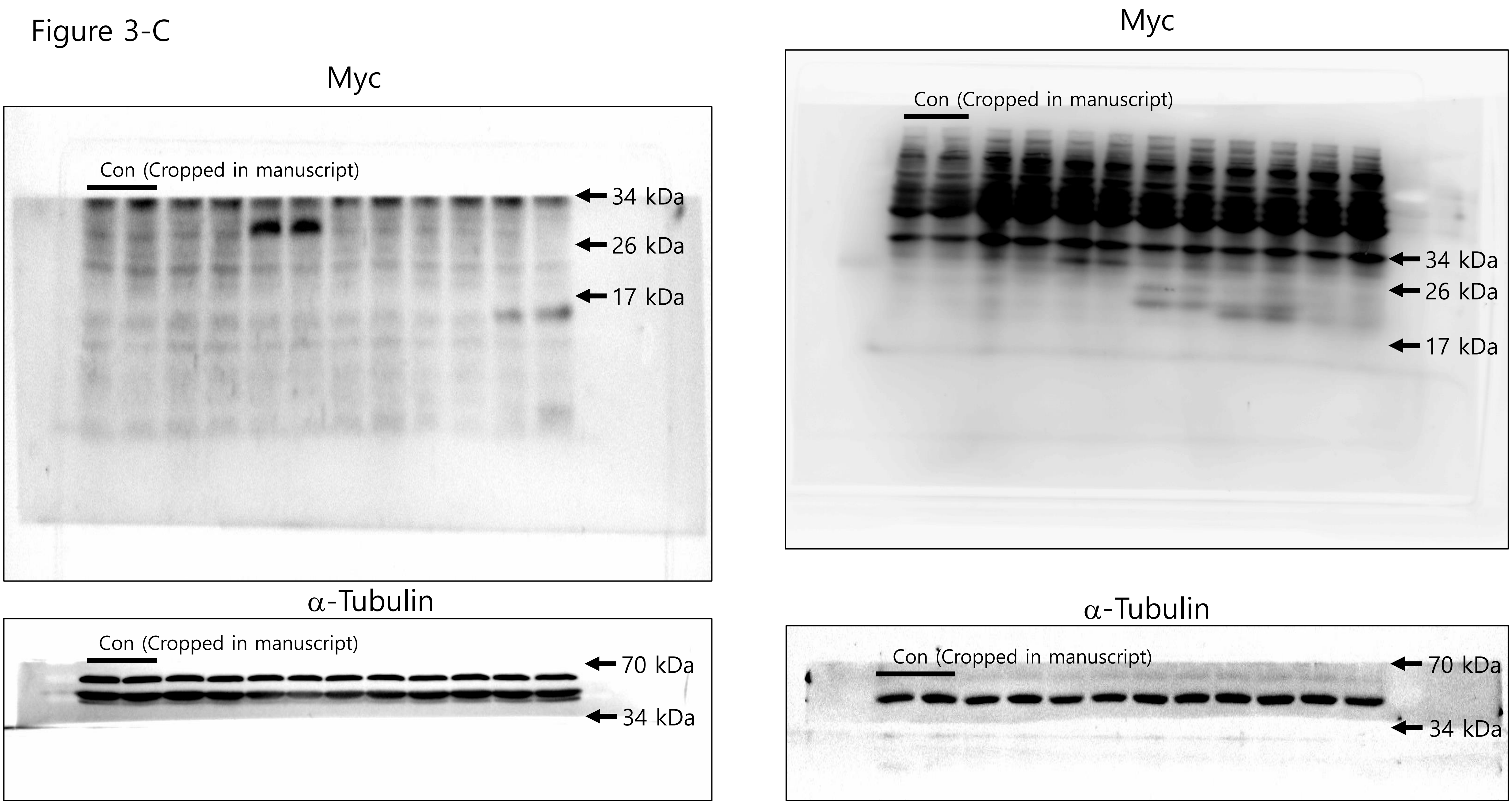
Myc
Con (Cropped in manuscript)
34 kDa
26 kDa
17 kDa
α-Tubulin
Con (Cropped in manuscript)
70 kDa
34 kDa
Myc
Con (Cropped in manuscript)
34 kDa
26 kDa
17 kDa
α-Tubulin
Con (Cropped in manuscript)
70 kDa
34 kDa

Figure 3-D

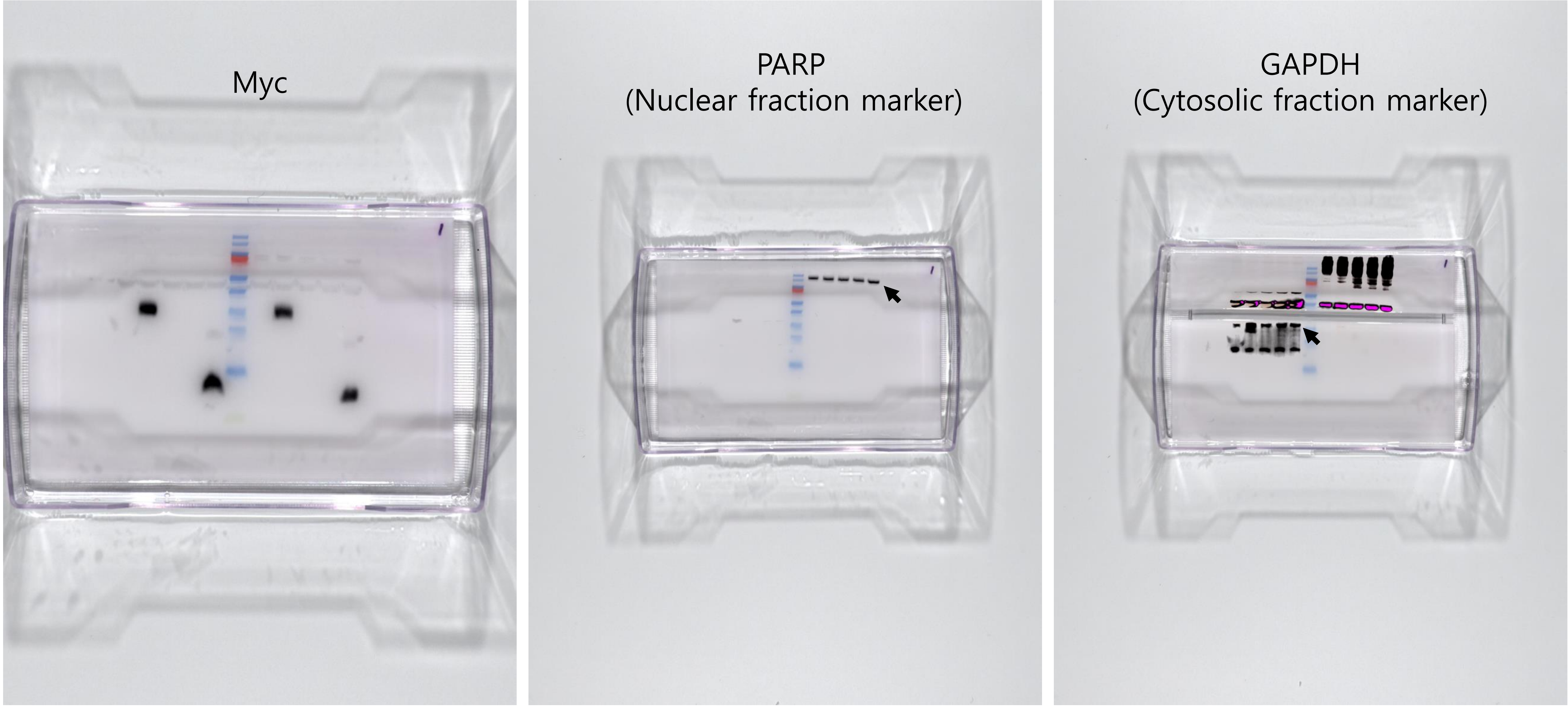
Myc
PARP
(Nuclear fraction marker)
GAPDH
(Cytosolic fraction marker)

Figure 3-E

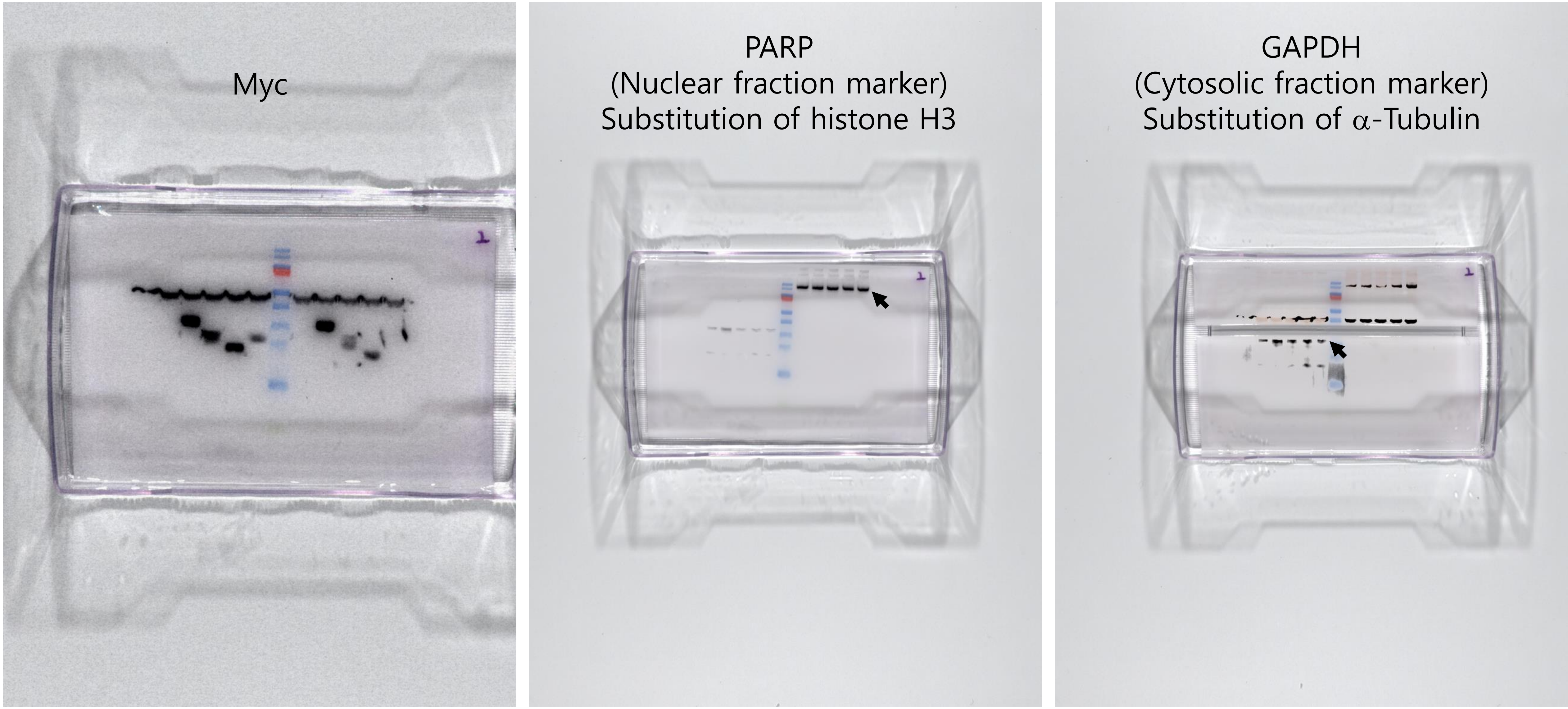
Myc
PARP
(Nuclear fraction marker)
Substitution of histone H3
GAPDH
(Cytosolic fraction marker)
Substitution of α-Tubulin

Figure 4-C

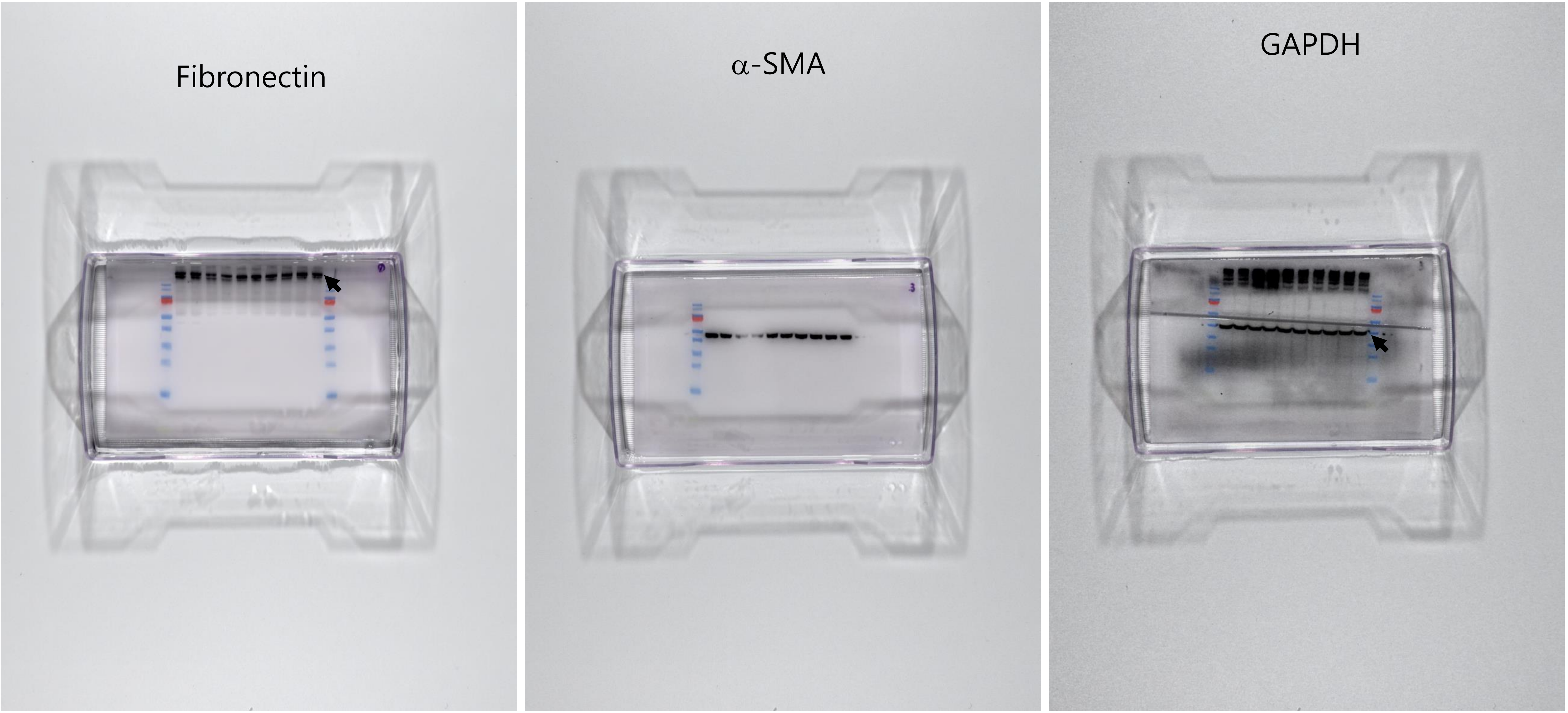

Figure 4-F

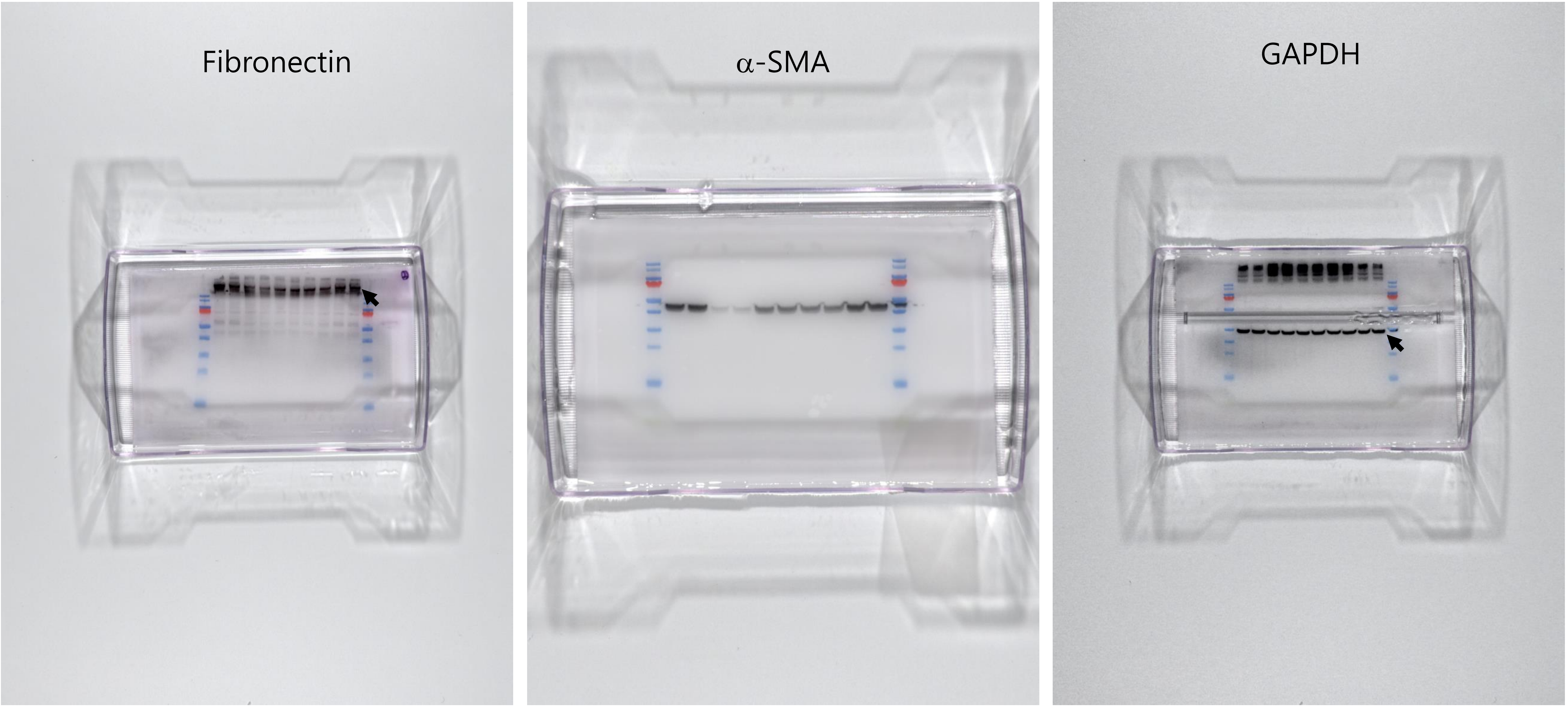

Supplementary Figure 2-C

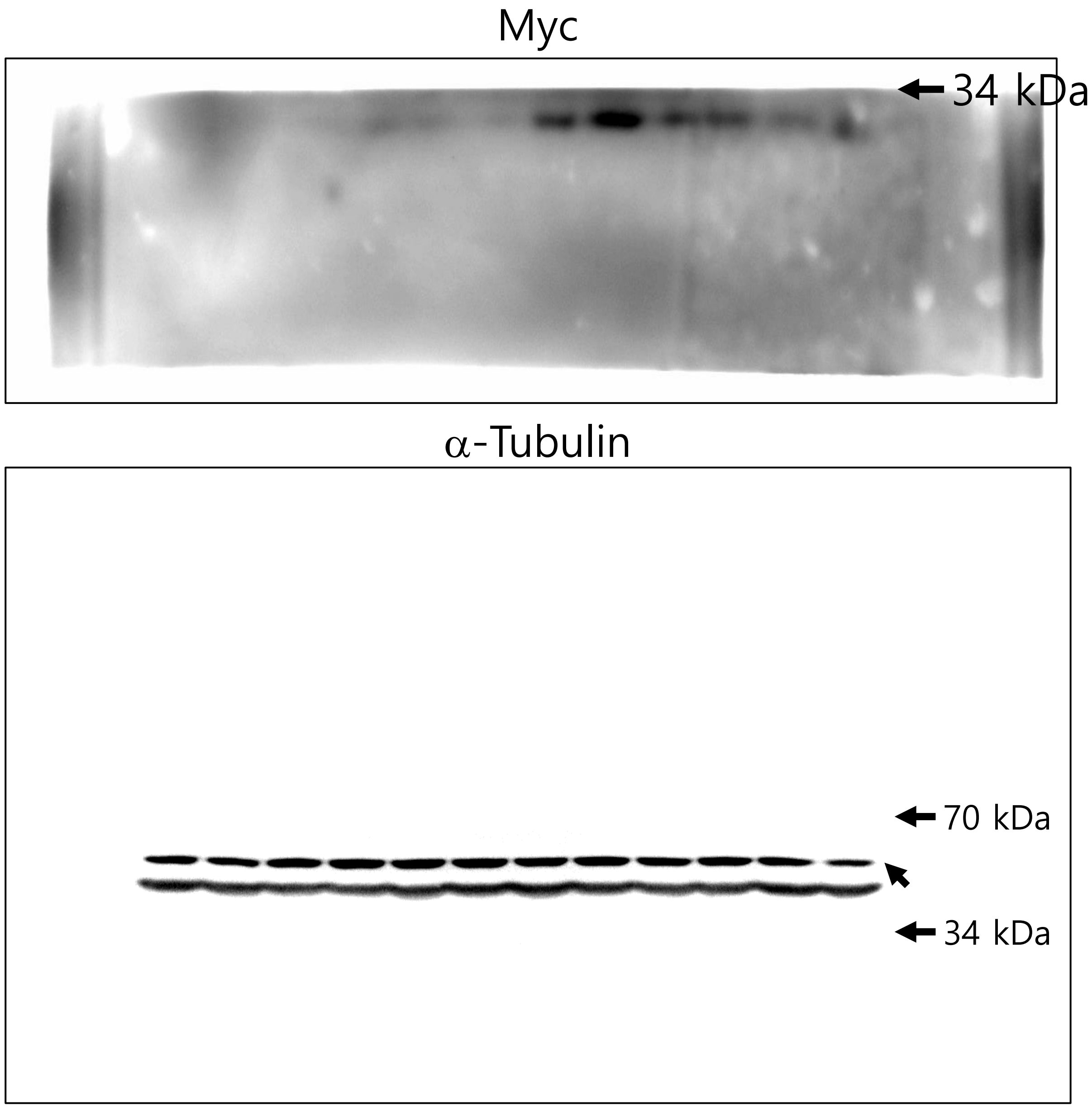

Supplementary Figure 3-C

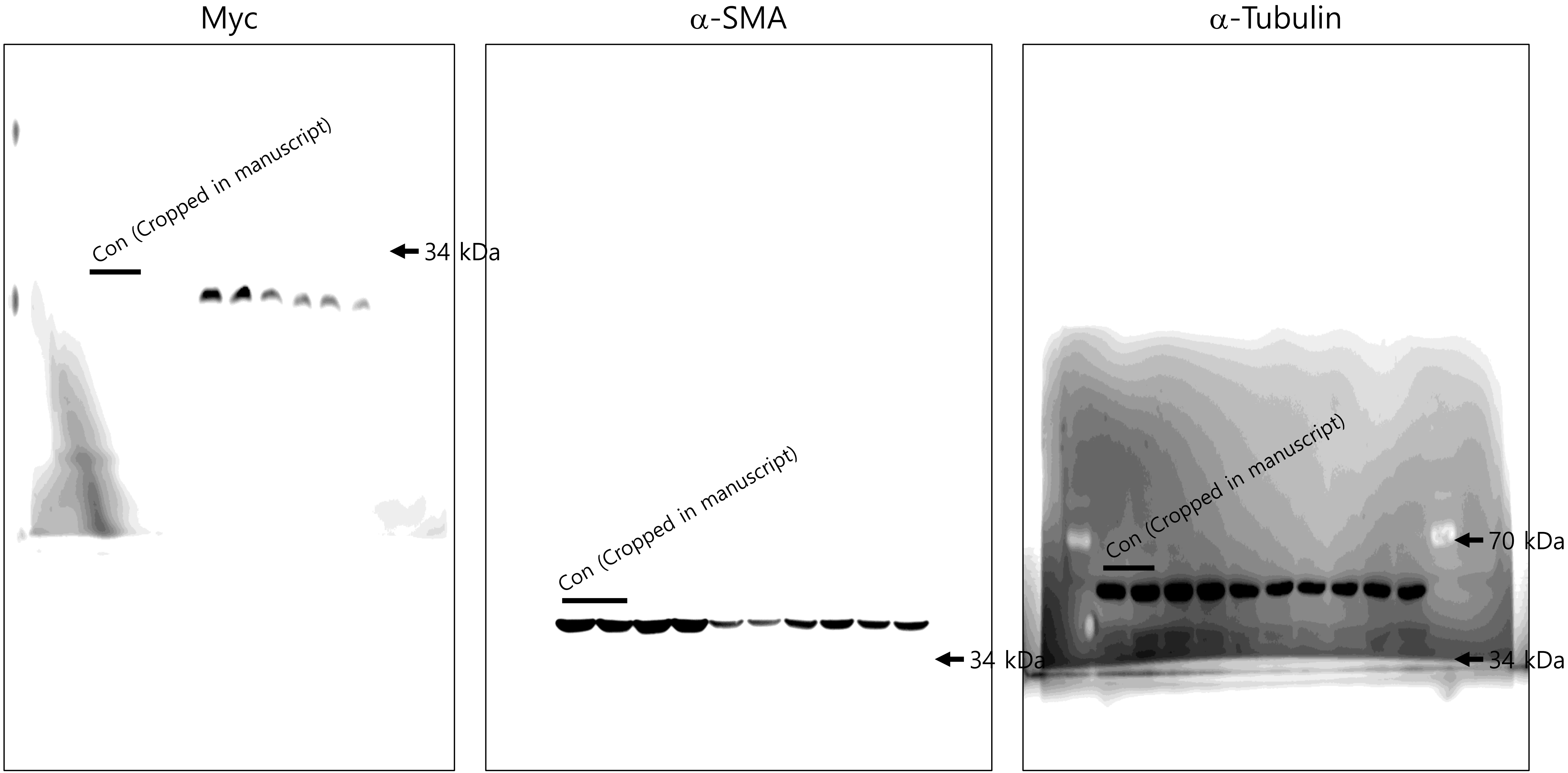

Supplement: S1 Raw images — (PDF) [file pone.0267629.s008.pdf]
